# Effectiveness of Radiofrequency Microneedling in the Treatment of Dermatological Conditions: A Systematic Review

**DOI:** 10.1007/s00266-026-05834-y

**Published:** 2026-04-28

**Authors:** Narendra Kumar, Hyoung Moon Kim, Ayaka Nishikawa, Chan Yeong Heo, Woffles T. L. Wu

**Affiliations:** 1 Global Medical Affairs, Jeisys Medical Inc., 806,96 Gamasan-ro, Geumcheon-gu, Seoul, 08501 Republic of Korea; 2 Maylin Clinic Ilsan, Goyang, Republic of Korea; 3 Cosmetic Dermatology, SBC Medical Group, Medical Corporation Shoubikai, Tokyo, Japan; 4 Department of Plastic and Reconstructive Surgery, Seoul National University, Bundang-gu, Seongnam-si, Gyeonggi-do 463-707 Republic of Korea; 5 Woffles Wu Aesthetic Surgery & Laser Centre, Singapore, Singapore

**Keywords:** RFMN, Radiofrequency Microneedling, Dermatological conditions, Skin condition, Efficacy, Safety, Patient-reported outcomes

## Abstract

**Background:**

Radiofrequency microneedling (RFMN) utilizes RF energy delivered via needles to the dermis to enhance texture, reduce laxity, and improve dyschromia. However, evidence has been fragmented by indication/device heterogeneity. We evaluated clinical effectiveness, safety, and patient-reported outcomes (PROs) of RFMN across dermatological conditions.

**Methods:**

Searches of PubMed, Google Scholar, and Semantic Scholar (January 2015–July 2025) (PROSPERO registration: CRD420251089393) were conducted, and English-language RCTs, cohort studies, case series, and reports of RFMN across skin indications were included (non-human studies excluded). A modified Critical Appraisal Skills Programme (CASP) checklist was used for quality analysis. Findings were narratively synthesized.

**Results:**

Forty-one studies met the criteria (15 RCTs, 19 prospective non-randomized studies, 5 prospective cohorts, 2 case series) spanning diverse geographies and Fitzpatrick skin types. For atrophic acne scars, RFMN consistently reduced scar scores and demonstrated efficacy comparable to fractional lasers. For skin laxity/photoaging, RFMN improved wrinkle scales, dermal density, and submental volume, (histologically evidenced). Additional benefits were noted for rosacea, melasma, and striae, though effect sizes varied. In primary axillary hyperhidrosis, *botulinum* toxin A outperformed RFMN for symptom reduction/comfort. Safety was favorable; erythema, edema, and transient pain predominated; post-inflammatory hyperpigmentation was infrequent and self-limited. PROs indicated high satisfaction and minimal downtime. Reporting of technical parameters, especially temperature, pulse width, and cooling, was inconsistent.

**Conclusions:**

RFMN is safe, well-tolerated, with credible efficacy across multiple indications and skin types, and an attractive recovery profile. Standardized outcomes, longer follow-up periods, and rigorous reporting of device settings are necessary to refine protocols and facilitate head-to-head device comparisons.

**Level of Evidence III:**

This journal requires that authors assign a level of evidence to each article. For a full description of these evidence-based medicine ratings, please refer to the Table of Contents or the online Instructions to Authors www.springer.com/00266.

**Supplementary Information:**

The online version contains supplementary material available at 10.1007/s00266-026-05834-y.

## Introduction

Radiofrequency (RF) technology has undergone significant evolution over the past two decades, originating from early monopolar and bipolar devices used for tissue tightening and coagulation. The introduction of fractional RF in the mid-2000s improved precision and reduced downtime. A significant advancement occurred with the integration of microneedling and RF, enabling direct intradermal energy delivery with enhanced collagen remodeling and improved safety. Radiofrequency microneedling (RFMN) was developed as a versatile and minimally invasive aesthetic treatment [1, 2]. It has emerged as a significant advancement in modern aesthetic and plastic surgery practice, offering a safe and effective adjunct to traditional surgical procedures [3]. This innovative dermatologic technique combines the benefits of microneedling with the targeted delivery of RF energy to the deeper layers of the skin. It enhances skin tightening and rejuvenation with minimal complications, beyond which is achieved by conventional microneedling [4, 5]. The procedure stimulates collagen and elastin production through controlled dermal injury, leading to skin remodeling and improved texture and tone [6]. Improvements in skin tissue are reported to be observed histologically within as short a timeframe as 4 days [3], with results continuing to enhance over several months and persisting up to 6 months post-treatment [5]. The ability to tailor treatment parameters for different facial regions further optimizes both safety and clinical outcomes. As a result, RFMN is increasingly recognized as a versatile and valuable tool in comprehensive aesthetic care [3]. It has shown effectiveness in treating various skin conditions, including acne scarring, skin laxity, striae, melasma, rosacea, cellulite, hyperhidrosis, and androgenetic alopecia [1, 5].

RFMN employs various modalities to optimize treatment precision and safety. Key modalities include monopolar or bipolar RF energy delivery, insulated or non-insulated needles, controlled pulse durations, and adjustable needle depths for targeted dermal penetration. Devices may vary in needle geometry and array design to suit specific indications. Advanced systems also incorporate real-time feedback (such as temperature or impedance) to enhance efficacy and minimize thermal injury [2, 7]. This technological versatility has led to the development of multiple commercial RFMN platforms, each designed with unique configurations to optimize clinical outcomes. Table 1 provides an overview of some of the prominent devices currently available worldwide.

**Table 1:** List of some of the currently marketed RF devices worldwide

Brand	Manufacturer	RF type	United States food and drug administration (US FDA) cleared (510(k))	FDA-cleared indication(s)
Bodytite	Invasix, Israel	Bipolar	No	–
Cosderma dual handle MNRF	Cosderma	Bipolar	No	–
Exion	BTL Aesthetics	Monopolar	Yes	Tissue heating for pain relief, muscle spasm reduction, and improved local circulation, along with dermatologic, and general surgical applications for electrocoagulation, hemostasis, and fractional skin treatment
Genius RF	Lutronic	Bipolar	Yes	Dermatologic procedures for electrocoagulation and hemostasis
INFINI	Kutronic Corp, Goyang, South Korea	Bipolar	Yes	Dermatologic and general surgical procedures for electrocoagulation and hemostasis, and the percutaneous treatment of facial wrinkles
Intensif applicator	EndyMed, Caesarea, Israel	Non-insulated fractional microneedle radiofrequency (NFMRF)	Yes	Dermatologic and general surgical procedures for electrocoagulation and hemostasis
Modern aesthetics MNRF	Modern aesthetics	Bipolar	No	–
Morpheus8	InMode	Bipolar	Yes	Dermatologic procedures for electrocoagulation, hemostasis, and soft-tissue coagulation
Pixel8 RF	Rohrer Aesthetics	Bipolar	Yes	Dermatologic procedures for soft-tissue coagulation and hemostasis
POTENZA	Jeisys Med Inc.	Monopolar & bipolar	Yes	Dermatologic and general surgical procedures for electrocoagulation and hemostasis
Profound RF	Candela	Bipolar	Yes	Dermatologic procedures including percutaneous electrocoagulation and hemostasis
Scarlet RF	Viol Co. Ltd.	Bipolar	Yes	Dermatologic and general surgical procedures for electrocoagulation and hemostasis
Secret RF	Cutera	Bipolar	Yes	Dermatologic and general surgical procedures for tissue coagulation and hemostasis
SylfirmX	Viol Co. Ltd.	Bipolar	Yes	Dermatologic procedure for electrocoagulation and hemostasis
United II	Shenzhen Peninsula Medical Co.	Negative pressure fractional microneedle radiofrequency	No	–
Virtue RF	Cartessa Aesthetics	Bipolar	Yes	Dermatologic procedures for electrocoagulation and hemostasis
Vivace	Aesthetics Biomedical	Bipolar	Yes	Dermatologic procedures for electrocoagulation and hemostasis

Multiple systematic literature reviews (SLRs) and randomized controlled trials (RCTs) have validated RFMN’s efficacy and safety in treating acne scars, hyperhidrosis, striae distensae, and skin laxity. However, despite the growing popularity of RFMN in dermatologic practice, existing literature is often fragmented, focusing on isolated indications such as acne scars or skin laxity, and lacking a uniform assessment of efficacy across broader dermatological conditions. Moreover, previous reports have failed to consistently evaluate key parameters such as treatment safety, adverse events, and patient-reported outcomes (PROs). There is currently no comprehensive systematic review that integrates clinical outcomes, device-specific variables, and patient-centered evidence across a range of skin conditions. This gap presents a challenge for dermatologists and plastic surgeons seeking evidence-based guidance on the optimal use of RFMN. Therefore, the present systematic literature review (SLR) aims to synthesize available evidence on the clinical effectiveness, safety profile, and PROs of RFMN across dermatological indications, while elucidating the impact of device-specific parameters on treatment outcomes to support evidence-based clinical decision-making and optimization of treatment protocols.

### Study Objective

This review aims to systematically:

- Evaluate the clinical effectiveness of RFMN across dermatological indications, including acne scars, skin laxity, photoaging, pigmentary disorders (e.g., melasma and hyperpigmentation), rosacea, striae, and hyperhidrosis.
- Characterize the safety profile of RFMN and adverse event spectrum by assessing the incidence, type, and severity of reported adverse events.
- Evaluate PROs, including treatment satisfaction, tolerability, downtime, and quality-of-life measures.
- Examine the influence of device-specific and procedural parameters—such as needle depth, energy settings, insulation type, and monopolar versus bipolar delivery modes on clinical outcomes and safety.
- Identify evidence gaps, sources of heterogeneity, and methodological limitations to inform future research priorities and support optimization of RFMN treatment protocols.

## Methods

This systematic review was conducted in accordance with a pre-specified protocol registered in PROSPERO (CRD420251089393) and reported by the Preferred Reporting Items for Systematic Reviews and Meta-Analysis (PRISMA) checklist [8]. To minimize selection bias, a predefined population, intervention, comparator, and outcome (PICO) framework, along with a two-reviewer screening process, was employed. To minimize reporting bias, comprehensive searches were performed in three databases—PubMed, Semantic Scholar, and Google Scholar. Google Scholar was included to cover gray literature, which helps mitigate reporting bias as well.

### Eligibility Criteria

#### Inclusion Criteria (PICO Framework)

*Population*: Individuals of any age or gender, diagnosed with dermatological conditions such as aging, facial rhytids, acne scars, cellulite, striae, rosacea, axillary hyperhidrosis, melasma, skin laxity, wrinkles, fine lines, and hyperpigmentation.

*Intervention*: RFMN, including fractional RFMN, bipolar or monopolar RF devices, insulated or non-insulated tips, any session frequency, and protocol.

*Comparator(s)*: Absent or present, including microneedling alone, surgical interventions, topical treatments, laser treatments, placebo/sham treatment, or no treatment.

*Outcomes*: *Primary*: Clinical outcomes include scar grading scales, pigmentation indices, skin elasticity, skin hydration, wrinkling, transdermal water loss, skin laxity, patient-reported outcomes, satisfaction, and tolerability.

*Secondary:* Recovery time, adverse effects such as erythema, pain, infection, edema, purpura, long-term efficacy, and any histological changes.

*Types of studies/data sources*: Randomized controlled trials, case series, case reports, observational studies, including cohort studies, case–control studies, and cross-sectional studies published in English or with English language translations, from January 2015 to July 2025, with no geographical restrictions.

#### Exclusion Criteria

Studies were excluded if they did not focus on dermatological conditions or if RFMN was not the primary intervention or was used solely as an adjunct without separate outcome reporting. Non-human or pre-clinical studies were also excluded, as were those lacking relevant clinical or patient-reported outcomes. Additionally, non-peer-reviewed sources, including theses, conference abstracts, editorials, and content from manufacturer websites, were not considered. Secondary research, such as systematic reviews, scoping reviews, umbrella reviews, and narrative reviews, was excluded.

#### Outcomes of Interest

Evaluated clinical outcomes of interest included scar grading scales, pigmentation indices, skin elasticity, hydration, and wrinkling, and any indication-specific efficacy parameter reported by the included studies. Patient-reported outcomes focus on measures such as patient-reported pain, tolerability, and satisfaction. Secondary outcomes included recovery time and adverse effects (e.g., erythema, infection), and any other indication-specific secondary outcomes reported, such as histological changes.

#### Data Sources and Search Strategy

A comprehensive literature search was conducted using PubMed, Semantic Scholar, and Google Scholar. Search terms were identified based on team discussions and preliminary scoping searches. Database search was limited to studies published between January 2015 and July 2025 to ensure the inclusion of the most relevant and up-to-date evidence.

To conduct the search, variations on the following search string were employed: (“microneedling radiofrequency” OR “radiofrequency microneedling” OR “fractional radiofrequency” OR “fractional radiofrequency microneedling” OR “fractional RF microneedling” OR “radiofrequency needling” OR “radiofrequency percutaneous collagen induction”) AND (“dermatological conditions” OR “acne scars” OR “melasma” OR “skin rejuvenation” OR “striae” OR “hyperpigmentation” OR “rosacea” OR “wrinkles”). Details on search strings according to the database are presented in Supplementary Table 1.

#### Study Selection

Duplicate references were removed from the query results using Zotero’s built-in duplicate detection feature. Screening was conducted in two stages using the Catchii online SLR tool [9]. In the first stage, one reviewer screened titles and abstracts against the predefined inclusion and exclusion criteria. Studies that did not meet the criteria were excluded, with reasons documented. In the second stage, full-text articles were reviewed independently by two reviewers. Discrepancies were resolved through discussion and consensus.

#### Data Extraction

Data were extracted from eligible studies by two independent reviewers using a pre-designed data extraction form in Microsoft Excel. The form was piloted on a random sample of three studies to ensure completeness and clarity. Extracted data included study characteristics (author, year, country), population details (age, sex, dermatological condition), intervention and comparator, technical details (needle depth, power settings, number of sittings), reported outcomes and results, and any AEs. Any conflicts were resolved by consensus.

#### Quality Assessment

The methodological quality of the included studies was assessed using a modified Critical Appraisal Skills Programme (CASP) checklist, which included items relevant to each study design. The CASP checklist assesses key domains, including study validity (including risk of bias), reliability, and the applicability of results [10]. Each study was rated based on individual quality criteria, and an overall quality rating was assigned. Study findings were interpreted with caution if the overall quality rating was not high. However, no studies were excluded based on the quality ratings. Quality assessment was conducted independently by two reviewers, with any disagreements resolved through consensus.

#### Data Synthesis

A qualitative synthesis approach was used to analyze and present the findings from the included studies. Due to anticipated heterogeneity in study designs, RFMN devices, treatment protocols, and dermatological conditions, a narrative synthesis was conducted rather than a quantitative meta-analysis. A detailed description of the included studies was developed in both text and tabular formats to facilitate comparison across studies. The synthesis was structured around clinical effectiveness, safety outcomes, patient satisfaction, and device characteristics. Device performance was compared across studies and indications.

## Results

**Fig. 1:**
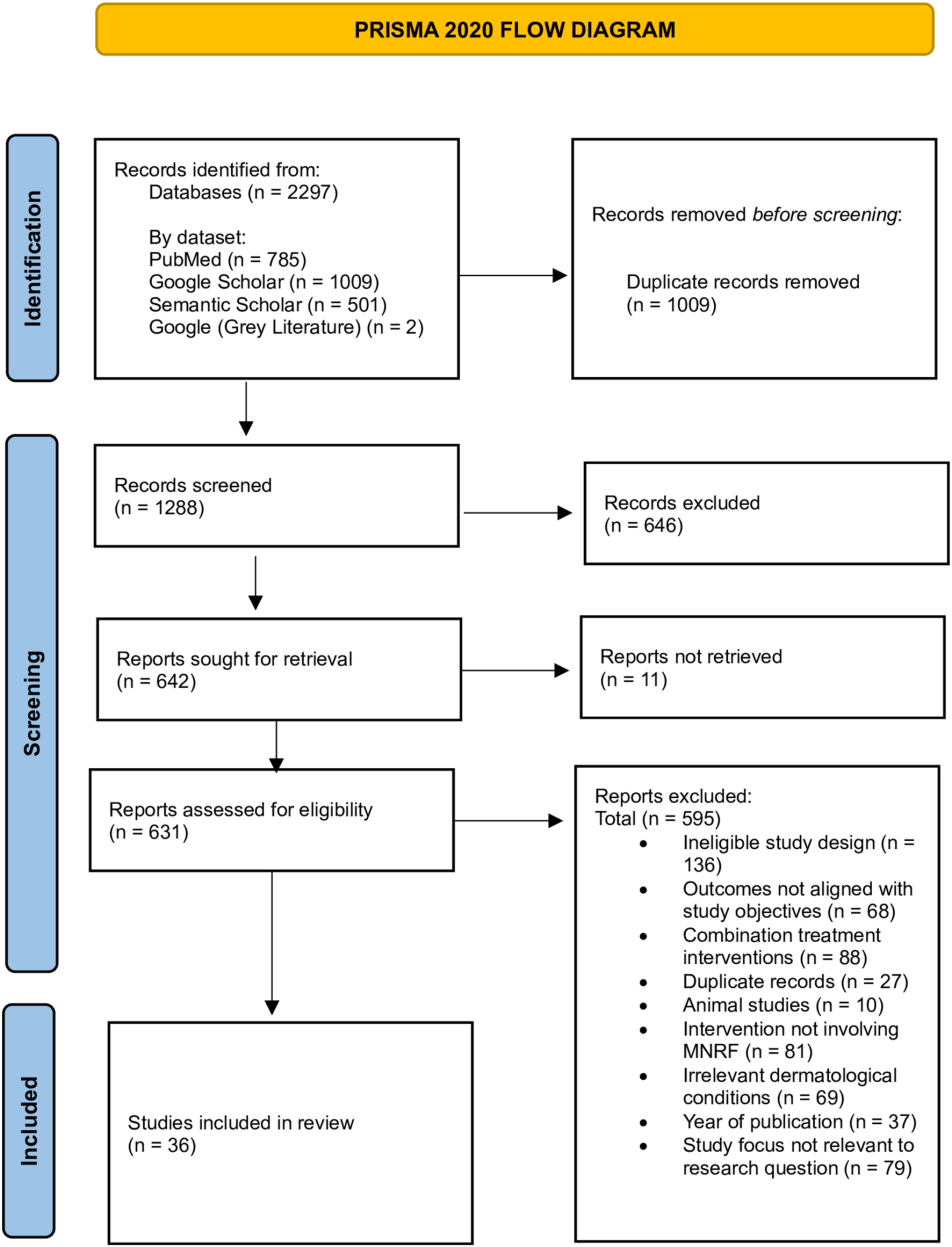
PRISMA flowchart

A total of 2371 records were identified from three databases (PubMed, Google Scholar, and Semantic Scholar); Figure 1 (PRISMA diagram) shows the approach to the selection of the studies in the systematic review.

**Fig. 2:**
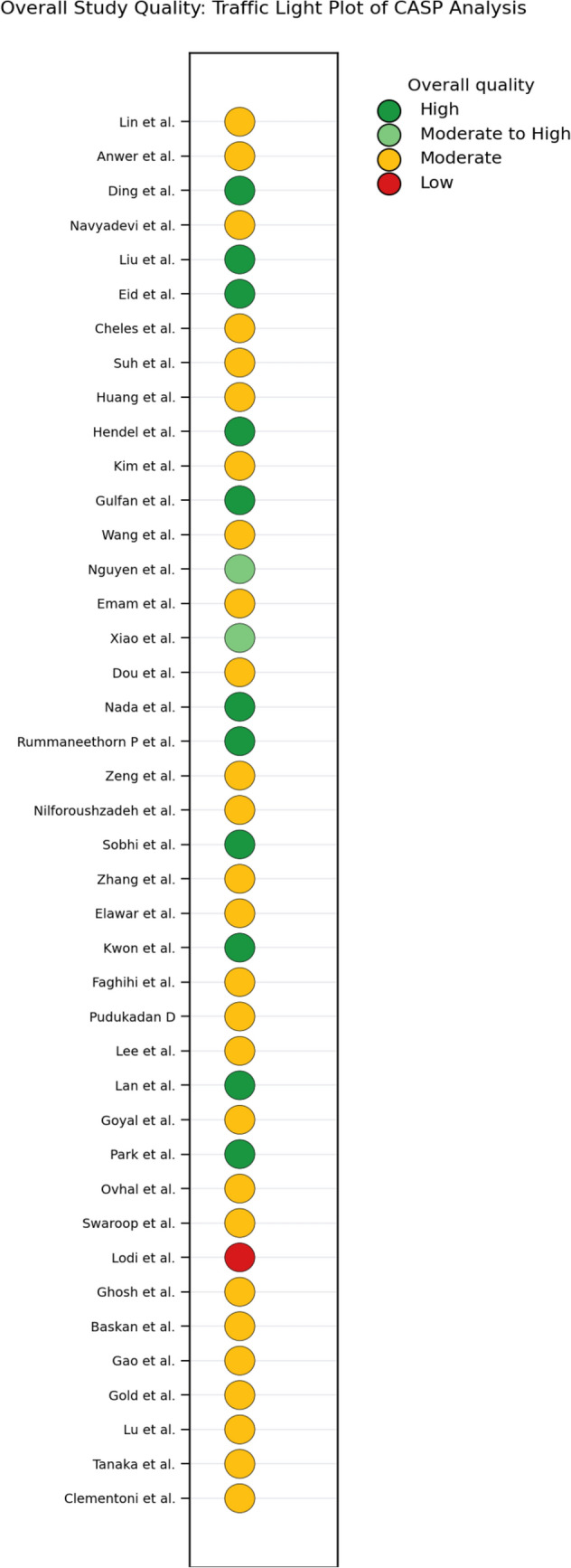
Quality assessment of included studies using the CASP checklist

The included studies (n = 41) were assessed for quality and risk of bias using a modified CASP checklist. The results are summarized in Figure 2, and detailed assessment data for each CASP domain are presented in Supplementary Table 2. Additionally, the certainty of evidence has been assessed for all the studies and presented according to indication, in Table 2. Overall, the studies demonstrated moderate-to-high methodological rigor, despite several limitations. Most studies addressed a clearly defined research question, though information on long-term efficacy and safety was limited.

**Table 2:** Indication and evidence strength to assess the certainty of evidence among the included studies (based on quality assessment)

Indications	Type of manuscript	Level of evidence	References
Periocular/periorbital wrinkles	Prospective, intraindividual-controlled, single-center study	Moderate	Lin et al. [11]
	Cohort study	Moderate	Kim [12]
	Prospective single-center study	Moderate	Cheles et al. [13]
	Prospective study	Moderate	Lee et al. [14]
	Prospective, intraindividual self‑controlled clinical trial	Moderate	Gao et al. [15]
Photoaging	Retrospective study	Moderate	Anwer et al. [16]
	Prospective split-face randomized controlled trial	Moderate	Dou et al. [17]
	Prospective study	Moderate	Zhang et al. [18]
Acne scars	Real world clinical study	High	Ding et al. [19]
	Retrospective Study	Moderate	Huang et al. [20]
	Prospective Interventional Study	Moderate	Navyadevi et al. [21]
	Randomized blinded split-face comparative study	High	Hendel et al. [22]
	Randomized split-face blinded trial	Moderate	Emam et al. [23]
	Cohort study	Moderate	Elawar and Dahan [24]
	Randomized split-face study	Moderate	Faghihi et al. [25]
	Cohort study	Moderate	Pudukadan [26]
	Two-center, prospective comparative clinical study with randomized split-face design	High	Lan et al. [27]
	Prospective comparative study	Moderate	Goyal et al. [28]
	Retrospective cohort study	Moderate	Ovhal [29]
	Retrospective study	Moderate	Baskan and Belli [30]
Face/neck rejuvenation	Retrospective chart review	Moderate	Suh et al. [31]
	Prospective intraindividual controlled study	Moderate to high	Nguyen et al. [32]
	Retrospective study	Moderate to high	Xiao et al. [33]
	Pretest/post-test clinical trial	Moderate	Nilforoushzadeh et al. [34]
	Prospective randomized split-face study	High	Liu et al. [35]
	Prospective multicenter clinical trial	Moderate	Gold et al. [36]
	Prospective, double-blind, randomized, controlled face-split study	Moderate	Lu et al. [37]
	Prospective interventional study with 3‑D volumetric analysis	Moderate	Tanaka et al. [38]
	Prospective pilot pretest/post-test study	Moderate	Clementoni et al. [39]
Bilateral facial melasma	Pilot study	High	Gulfan et al. [40]
Rosacea	Prospective observational study	Moderate	Wang et al. [41]
	Prospective, randomized, split-face clinical trial	High	Park et al. [42]
Striae distensae	Cohort study	High	Nada et al. [43]
	Cohort study	High	Sobhi et al. [44]
	Cohort study	Moderate	Swaroop et al. [45]
	Randomized controlled study	Moderate	Ghosh [46]
Axillary hyperhidrosis	Prospective, randomized, assessor-blinded, intraindividual split side comparative study	High	Rummaneethorn and Chalermchai [47]
	Randomized controlled trial	High	Eid et al. [48]
Acne vulgaris	Prospective, single-blind, randomized, comparative clinical trial with a split-face design	Moderate	Zeng et al. [49]
	Prospective, randomized split-face study	High	Kwon et al. [50]
	Cohort study (Case series)	Low	Lodi et al. [51]

**Fig. 3:**
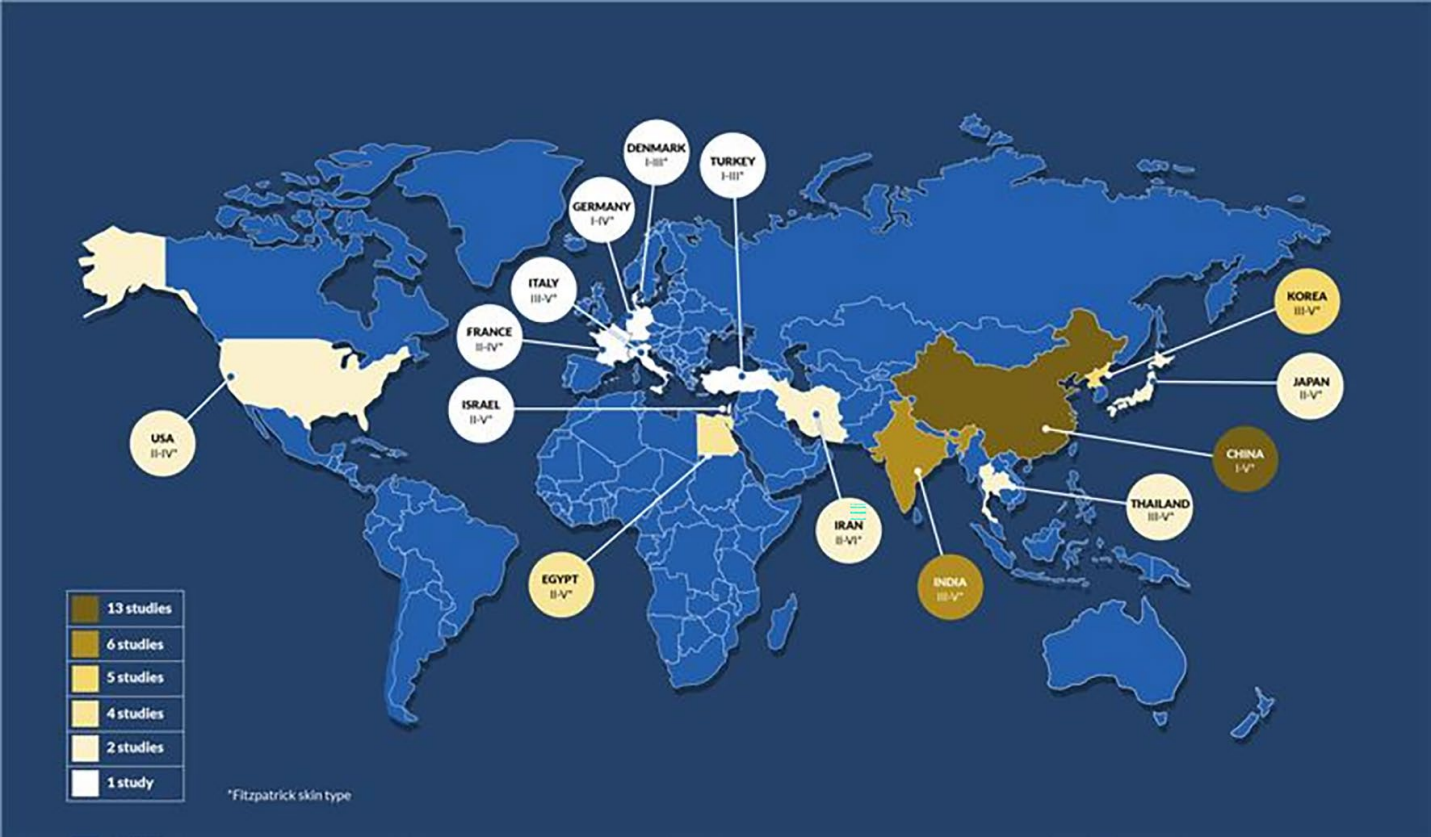
World map showing the geographical distribution of the included studies. The number of studies from each country and the Fitzpatric skin types included in the studies are mentioned for each country

Confounding factors and sources of bias were partially addressed in many studies. Several studies employed retrospective study designs [16, 52], which may be associated with limitations, such as selection bias. Across all studies, as Figure 2 shows, CASP scoring revealed that one study was rated as low quality, 13 (approximately 32%) were rated as high quality, and the remaining studies were rated as moderate-to-high or moderate with some concerns. The study characteristics are summarized in Table 3. Of the 41 studies, 15 were randomized clinical trials, 19 were prospective non-randomized studies, 5 were retrospective cohort studies, and two were case series. Study settings also varied from dermatology clinics and outpatient centers [16] to dermatology departments attached to medical university hospitals or academic dermatology centers. The study spanned a wide geography including Europe (France, Italy, Denmark, Germany, and Turkey, 1 each), United States (US) (2 studies—1 only in the US, and the other with an additional center in Japan), Asia (China, 13; Thailand, 2; Japan, 1 (another study was conducted in the US and Japan); Korea/South Korea, 5; India, 6), and Middle East (Israel, 1; Iran, 2; Egypt, 4), ensuring the inclusion of a diverse population. The countries and the number of studies along with the Fitzpatrick skin type covered in those studies are depicted in Figure 3.

**Table 3:** Comprehensive summary of study characteristics

Study	Design	N	Country	Indication	Device	Depth	Power	Needle Type	Pulse duration	Temperature	Sessions	Comparator	Outcomes	Follow-up	Adverse events	Cooling strategy
Lin et al. [11]	Prospective, intraindividual-controlled, single-center study	18	China	Periocular Static Wrinkles	MicroRF9 (Shenzhen Peninsula Medical Co. Ltd., China)	0.5–1.5 mm	4.0–10.0 W	Non-insulated microneedle electrodes/pins	200–300 ms	–	2	–	All patients experienced pain during MNRF^a^ In terms of perceived improvement, 11.1% felt very much improved, and 55.6% much improved; 33.3% improved.	6 months	Patients reported pain with MNRF^a^ (severe pain (22.2%), moderate pain (33.3%), mild pain (44.4%). Post-surgery transient erythema, edema, pain (100%), postoperative bruising (5.88%), and postoperative bleeding (29.41%) were also observed.	Cooling of skin with saline gauze
Anwer et al. [16]	Retrospective Study	96	China	Photoaging wrinkles	Intensif (EndyMed Medical, Israel)	1.7–2.7 mm	12–17 W	Non-insulated microneedle	80–140 ms	–	1–3		Almost all subjects reported pain (pain score 3 to 4), but the pain was described as bearable.	Repeated after 3 months	Patients reported erythema and bearable pain (100%) with MNRF^a^; no other reactions like scabs, swelling, and pigmentation were reported.	
Ding et al. [19]	Real World Clinical Study	126	China	Atrophic Acne Scars	FMR 49, (Shenzhen Peninsula Medical Co., Ltd, China)	1.6–2.0 mm	6–16 W	Insulated/non-insulated microneedle	200–500 ms	–	3		Average pain score of ~2.5/10 reported by the patients.	3–14 months	Patients reported immediate redness, swelling, heat, and mild-to-moderate pain (100%) (average score: 3.4); grid-like erythema (4.8%), and post-inflammatory hyperpigmentation (1.1%) were also observed.	
Navyadevi et al. [21]	Prospective Interventional Study	44	India	Acne Scars	Lumenis Legend PRO (Lumenis, Israel)	0.6–1 mm		Ultra-thin non-insulated needles	400 ms	–	4	–	Not reported	1-month	Patients reported erythema (100%), edema (27%), and post-inflammatory hyperpigmentation were also observed in 5% of patients.	Ice cubes prior to procedure
Liu et al. [35]	Prospective randomized split-face study	21	China	Facial tightening/wrinkles	Gold Microneedle United II (Peninsula, China)	1.5–3 mm	6 and 12 W	Non-insulated tip and insulated needle body	100, 300, and 600 ms	–	3	–	The differences in the VAS^e^ scores of the treated sides and control sides were 4.51 ± 1.38, 5.87 ± 1.28, 5.80 ± 1.09 at 1, 3, and 6 months, respectively.	6 months	Patients reported mild erythema, purpura, and heat sensation (100%).	After the intervention, the treated areas were cooled with ice packs (cool mask/cool gel) for 20 min.
Eid et al. [48]	Randomized Controlled Trial	20	Egypt	Primary axillary hyperhidrosis	Vivace® device (Shenb Co., Korea)	3.5 mm		Non-insulated needles	600 ms	–	3	Botox 1 session	Patients reported significantly higher satisfaction with MNRF^a^ than BT-A at all follow-ups: p ≤ 0.001. Pain was higher with FMR (mean 6.48) than BT-A (mean 4.00), p < 0.001.	3, 6, and 12 months	Significantly higher mean pain score in FMR versus BT-A group (6.48 ± 0.93, 95% CI: 6.04–6.91 versus 4.00 ± 1.75, 95% CI: 3.18–4.82, p < 0.001). Compensatory hyperhidrosis manifested in 2 patients on the same side treated with BT-A	–
Cheles et al. [13]	Prospective Single center Study	24	Israel	Periorbital wrinkles	Voluderm RFMN (Pollogen, Israel)	1 mm or 0.6 mm		Non-insulated ultrathin microelectrodes	–	–	4	–	Patients reported mild discomfort (mean VAS^e^ 4.1). 71% rated periorbital wrinkles as “improved” or “very much improved,” and 83% reported satisfaction with comfort, recovery, and results.	3 month	Patients reported mild edema, erythema, and itching (100%). Mild discomfort was also observed in patients.	
Suh et al. [31]	Retrospective Chart review	15	Korea	Facial rejuvenation	Profound system (Candela Medical, South Korea)		–	Insulated	3 Sec	67º	1	–	At the 4-month follow-up, 86.7% of patients reported satisfactory results, 73.4% were greatly satisfied, and 13.3 were satisfied	4 months	Patients reported pinpoint bleeding, mild-to-moderate erythema and edema (100%). Other serious AEs like hypo-or hyperpigmentation, skin burn, bullae or scarring were not observed.	
Huang et al. [20]	Retrospective Study	40	China	Atrophic Acne Scars	Bodytite, (Chongqing Peninsula Medical Technology Co. Ltd, China)	1–2 mm	5–6 W	Adjustable microneedles	400–600 ms	–	3	–	Patients reported mild, tolerable pain during MNRF treatment (VAS^e^ 1–4, mean 3.08).	3	Patients reported local mesh marks (4%), that disappeared after a week. Peri-oral herpes simplex was also reported (3%). Other AEs^b^ such as persistent hyperpigmentation or scar were not observed.	Ice was applied to treatment area
Hendel et al. [22]	Randomized blinded split-face comparative study	15	Denmark	Acne Scars	Genius RF (Lutronic, South Korea)	1.5–3.0 mm	30–98 mJ/pin	Bipolar microneedles	5–15%	–	1	Ablative fractional CO2 laser	Pain: RF (mean 3.2/10) vs. CO₂‚ laser (mean 5.6/10).	1- and 3- month	Patients reported hyperpigmentation (7%). None of the patients reported pain or discomfort during follow-up.	Cooled air
Kim [12]	Cohort Study	11	Korea	Periorbital Skin rejuvenation	Genius RF (Lutronic, South Korea)	1.8 mm	60 mJ/pin	Insulated fractional microneedles	–	–	3	–	All patients exhibited wrinkle improvement, Decrease in melanin content. Patient satisfaction varied from very satisfied (4), five satisfied (3), to three were slightly (3).	6-months	Patients reported no serious AE’s and pain. Erythema (11%) wrinkles and festooned eyebags at lower eye lids (22%) were observed.	–
Gulfan et al. [40]	Pilot Study	30	Thailand	Bilateral Facial Melasma	SYLFIRM (Viol, Korea)	0.8–1.5 mm	–	Non-insulated stainless steel microneedles	2MHz		3		Participants tolerated treatment well, with a reported mean pain score of 2.0 ± 1.8 (minimum, 0; maximum, 6).	6 months	Patients reported mild tolerable pain and mild transient erythema (100%). Recurrence of melasma was also observed (10%).	–
Wang et al. [41]	Prospective, observational study	35	China	Difficult-to-treat rosacea (erythema or papules)	Intensif applicator (EndyMed Medical, Caesarea, Israel)	2.5 mm	6–12 W	Non-insulated fractional microneedle RF(NFMRF)	110 ms	–	3	None	HB significantly decreased, visible improvements, efficacy for difficult-to-treat rosacea	24 weeks	Minor, transient burning sensations and worsening of erythema	
Nguyen et al. [32]	Prospective Intraindividual Controlled Study	30	Germany	Skin rejuvenation	Genius RF (Lutronic, South Korea)	–	18–50mJ/pin	Insulated microneedles		–	3	–	Most patients were very (65%) or mainly (95%) satisfied. All patients would recommend the treatment and reported minimal downtime.	3–6 months	Patients reported mild-to-moderate edema and erythema (100%). Itching and perioral dermatitis with pustules (13.3%) and hematoma at the orbital rim (3.33%) were also reported. No other serious AE^b^ were observed.	
Emam et al. [23]	Randomized Split-face blinded trial	21	Egypt	Atrophic Post Acne Scars	Vivace, (Seoul,, Korea)	0.8–2.5 mm	–	Non-insulated microneedles (gold-coated)	600 ms	–	4	–	Downtime was shorter with MRF^a^ (≤2 days) versus Er: YAG (3_7 days)	3 months	Patients reported folliculitis (23.8%), erythema, eczema formation, and post-inflammatory hyperpigmentation (14.2%).	Post-procedure skin cooling with ice packs for 5–10 minutes w
Xiao et al. [33]	Retrospective Study	98	China	Neck Rejuvenation	Intensif (EndyMed Medical, Israel)	1.8–2.5 mm	12–14 W	Non-insulated gold-plated microneedles	80–100 ms	–	1–3	–	Almost all subjects reported 3 to 4 points of pain scores.	3 months	Patients reported erythema, scabs and pain (100%).	
Dou et al. [17]	Prospective split-face randomized controlled trial	15	China	Facial Photoaging	INFINITM (Lutronic Co., Korea)	0.5–1.5 mm	–	Electrode needle tip	50–150 ms	–	3	–	At 1- and 3-month post-treatment, 73–80% of MNRF^a^ patients and 66–73% of NAFL^i^ patients reported >50% satisfaction with skin laxity/photoaging.	3-month follow-up	Patients reported pain and scab crusting (100%)	–
Nada et al., [43]	Cohort Study	20	Egypt	Striae distensae	RF™; (Hanz, Beijing, China)	3 mm	4–5W	Non-insulated needles	300–400 ms	–	4	–	MNRF^a^ treatment (average 4.23) was nonsignificantly considered more painful than Er: YAG (average 3.41).		No significant side effects in MNRF‐treated sites.	–
Rummaneethorn et al. [47]	Prospective, randomized, assessor-blinded, intraindividual split side comparative study	20	Thailand	Axillary hyperhiderosis	FMR DeAge EX® applicator device (Daeshin Enterprise Co., Ltd., South Korea)	3.0–3.5 mm		Insulated microneedles	10–40 ms	–	2	Intradermal botulinum toxin Type A	Slightly higher VAS^e^ pain scores for microneedle RF vs. botulinum toxin A (3.45 vs. 2.65).	3 months	Patients reported mild prolonged erythema (10%), skin desquamation (5%), skin dryness (5%)	–
Zeng et al. [49]	Prospective, single-blind, randomized, comparative clinical trial with a split-face design	10	China	Acne vulgaris	INTRAcel (Jeisys, Korea)	0.8–2.0 mm	12.5 W	Partially insulated microneedles	80 ms	–	3	–	Patient satisfaction was higher for MNRF^a^ after the first treatment (8.1 ± 1.2 vs. 6.4 ± 1.0; p< 0.05).	3-month follow-up	The comparative analysis showed no significant difference between the pain scores on each side of the patient’s face.	Postoperative facial cooling mask (5% boric acid) for 15–20 mins
Nilforoushzadeh et al. [34]	Pretest/post-test clinical trial	20	Iran	Skin lifting/tightening	INFINI RF (Lutronic Corp, South Korea)	0.5–2.5 mm	5–10W	Stainless gold microneedles	100 ms	23 ºC (40% humidity)	6	–	At three months after the last session, patient satisfaction was significant (p< 0.05).	6-month follow-up	Patients reported mild erythema and edema (100%)	–
Sobhi et al. [44]	Cohort Study	17	Egypt	Striae distensae	Vivace Microneedling Device (Vivace, Korea)	3.5 mm	20 W	Non-insulated microneedles	400 ms	–	5	–	Patient were more satisfied in area B (fractional CO₂ laser)		Patients reported satisfactory improvement (good in 52.9%, very good in 5.88%, excellent in 5.88%, minimal in 35.2%). Post-inflammatory hyperpigmentation (52.9%) was also observed	Ice packs applied immediately
Zhang et al. [18]	Prospective Study	27	China	Facial Photoaging	INTRAcel (Jeisys, Korea)	0.5–0.8 mm	12.5 W	Microneedle electrodes	50–80 ms	–	3	–	Patients reported lower pain with unipolar RF (VAS^e^ 3.19 ± 1.77) compared to bipolar RF (VAS^e^ 5.63 ± 1.78)	6-month follow-up	Patients reported mild-to-moderate pain, mild bleeding, erythema and edema (100%). Pin-point purpura was observed in 81% of patients. None of the patients reported AEs^b^ such as hypo- or hyperpigmentation or aggravation of melasma.	–
Elawar and Dahan, [24]	Cohort Study	19	France	Atrophic acne scars	Intensif (EndyMed Medical, Israel)	2–3.5 mm	12–18 W	Non-insulated microneedle	110–140 ms	–	2–6	–	The average pain assessment score was 1.5 (0–10). Patients reported overall satisfaction and improvement with the treatment	3-month follow-up	Patients reported mild-to-moderate erythema and edema (100%). Other AEs such as hypo- or hyper pigmentation, burns or scars were not observed.	–
Kwon et al. [50]	Prospective, randomized split-face Study	25	Korea	Acne vulgaris	Secret (Ilooda, Korea)	0.8–2.0 mm	–	Microneedles	50–100 ms		3	Non-ablative 1450-nm diode laser	At the final 12-week follow-up, acne lesion counts decreased by 39.3% in the laser side, and 58.2% in the FMR side. No significant differences in downtime/impact on daily life.	3 months	No significant difference for post-treatment erythema and edema between two sides. PIH seen with MNRF in 2 patients. No scars or infection reported.	Cooling mask with soothing aloe gel
Faghihi et al. [25]	Randomized split-face Study	25	Iran	Facial acne scars	INFINI RF (Lutronic Corp, South Korea)	1.5–3.5 mm	50 W	Insulated microneedles	120–140 ms	–	3	Fractionated MNRF with and without subcision	Mean patient satisfaction VAS score was 5.1 ± 1.6 for MNRF^a^ without subscision and 6 ± 2.3 for MNRF with subscision	3 months	Most patients reported pinpoint bleeding and erythema. No other serious AE^b^ like scar formation, infection or ulceration, was reported. Transient bilateral submandibular lymphadenopathy transient PIH were also observed in 1 patient each.	Blood oozing was controlled with pressure and ice application.
Pudukadan [26]	Cohort Study	19	India	Atrophic acne scars	EndyMed PRO; Intensif handpiece (EndyMed Medical, Israel)	2.0–3.0 mm	15–25 W	Non-insulated gold-plated microneedles	110–140 ms	–	3	–	Improvement of at least 1 acne scar grade was noted in 11 of 19 patients (57.9%) after 1 month and in 9 of 9 patients (100%) after 3 months	3 months	Patients reported erythema (no to mild, 60%; moderate, 29.5% and significant, 10.5%), edema (no to mild, 65.3%). Dyschromia improvement (47.4%), post-inflammatory hyperpigmentation (5.3%) was also reported. In addition, the appearance of micro crusting was observed in some patients, while no other AE^b^ or infection was reported.	–
Lee et al. [14]	Prospective Study	20	Korea	Periorbital wrinkles	INTRAcel (Jeisys, Korea)	0.8 mm	12.5 W	Microneedle electrode	100 ms	–	3	–	Mean participant satisfaction score for the procedure was 3.05.	6-month follow-up	Patients reported mild erythema and edema (100%). Superficial Crusting was observed subsequently disappeared without scarring in 7 days.	Cooled with ice-packs post-treatment
Lan et al. [27]	Two-center, prospective comparative clinical study with randomized split-face design	60	China	Atrophic Acne Scars	Intensif (EndyMed Medical, Israel)	1.5–2.5 mm	10–15 W	Non-insulated gold-plated microneedle	80–140 ms	–	3	Accent microplasma RF revice	6 (10.00%), 25 (41.67%), 24 (40.00%), and 5 (8.33%) patients were clinically rated as showing excellent, good, moderate, and mild, improvement respectively, on the fractional MNRF side. More than 70% patients were satisfied in the fractional MNRF^a^ group.	6-month follow-up	Patients reported pain, erythema, and swelling (100%); post-inflammatory hyperpigmentation was not observed with fractional MNRF.	–
Goyal et al. [28]	Prospective, open-labeled comparative Study	33	India	Atrophic facial acne scars	MicroFrxcl (Derma, India)	–	–	–	–	–	3	EL1550 nm Erbium laser	Mean patient satisfaction steadily increased over visits, reaching 1.60 for MNRF^a^ and 1.64 for EL1550 nm laser at the final visit.	-	Patients reported edema (81.8%) and erythema (78.8%); post-inflammatory hyperpigmentation was observed in 5 patients and out of which 3 showed persistent needle marks.	–
Park et al. [42]	Prospective, randomized, split-face clinical trial	21	Korea	Rosacea	INFINI RF (Lutronic Corp, South Korea)	–	5–7.5 W	Insulated microneedles	50–70 ms	–	2	Untreated side	Clinical improvement of rosacea was observed in 17 (81.0%) of 21 total patients on the FMR-treated side. Satisfaction scores increased over time, indicating progressive patient-reported improvement in 12 weeks.	3 months	Patients reported mild pain (90.5%) and erythema (81%) for 3–5 days; residual pain and erythema were not observed in any patient.	–
Ovhal and Ovhal [29]	Retrospective Cohort Study	31	India	Acne Scars	Dermatrix (GSD, India)	–	11–142 mJ/pinand 20 J by assembly of pins	Electrodes (pin array)	–	–	3	–	Nearly all patients reported burning pain lasting 1–2 hours after treatment	–	Patients reported mild pain and erythema (100%). None of the patients reported post-inflammatory hyperpigmentation, while persistent track marks were observed in 1 patient.	–
Swaroop et al. [45]	Cohort Study	20	India	Striae distensae	DERMA INDIA (MR 16-2SB, India)	1.5–2.5 mm	50 W	Gold-plated insulated microneedle electrodes	450 ms	–	4	–	6 out of the twenty patients were ‘very satisfied’ with the treatment, 8 were ‘satisfied’ and 6 were ‘slightly satisfied’.	–	Few patients reported erythema, while no other AEs^b^ were reported.	Post-procedure, the sites were wiped gently with cold water and an ice pack was applied for 5 min
Lodi et al. [51]	Case Series	21	Italy	Acne vulgaris	Vivace; Deka M.E.L.A, Calenzano, Italy).	1400–1600 mm	4–5	Insulated needles with gold tips	400–500 ms	–	3	–	Patients experienced no pain or discomfort following the MNRF^a^ treatment and less downtime vs traditional monopolar/bipolar RF therapy.	6-month follow-up	Few patients reported erythema, while no other AEs^b^ were reported.	Ice packs immediately post-treatment
Ghosh et al. [46]	Randomized controlled study	30	India	Striae distensae	DERMA INDIA (MR 16-2SB, India)	0.5–3 mm	50 W	Gold-plated microneedles	450 ms	–	4	Fractional CO_2_ laser	The mean patient satisfaction VAS^e^ Score after treatment was 2.1 in Group B (MNRF^a^) as compared to 1.8 in Group A (Fractional CO₂).	–	Patients reported transient edema and erythema (100%), pain (13.33%). Other AEs^b^, like infection, ulcer scar or burn were not observed.	Ice pack was applied for 5 mins post-procedure.
Baskan and Belli [30]	Retrospective study	09	Turkey	Atrophic acne facial scars	Secret 2.0 (Ilooda, Korea)	2.5–3.5 mm	50–70 W	Insulated microneedles	RF conduction time :250–300 ms), needle conduction time: 450–550 ms).	–	1-5	–	Temporary pain was reported by 100% of patients, with the most common VAS^e^ score noted to be 1	3 months	There were no severe side effects, but temporary pain (100%; mode of visual analog scale, 1), erythema (100%), edema (100%), and crusting (11.1%) were reported.	–
Gao et al. [15]	Self-controlled clinical trial	25	China	Periorbital aging	High-frequency electrocautery, (United II, Shenzhan Peninsula Medical CO.)	1.1–1.5 mm	4–6 W	MicroRF negative pressure handle	100–330 ms	–	2	None	Periorbital wrinkles were significantly improved. Periorbital dermal thickness of the patients significantly increased	6 months	No adverse events. 1 patient developed PIH at 1 month after the first treatment	–
Lu et al. [37]	Prospective, randomized, controlled face-split study	13	China	Periorbicular and deep nasolabial wrinkles	Bodytite (Invasix, Israel)	0.5–2mm	12.5 W	49 microneedle electrodes	130 ms	–	3	None	Improvement of nasolabial folds in deep dermal approach	1 year	Punctuated scab	Postinterventional cooling with ice packs for 20 min
Gold et al. [36]	Prospective multicenter clinical trial	49	USA and Japan	Wrinkle	Endymed Pro Intensif applicator (EndyMed Medical, Caesarea, Israel)	1.3–2.8 mm	10–20 W	Non-insulated microneedle	80–140 ms	–	3	None	Improvement shown in 100% of patients with 65% of patients had significant improvement.	3 months	Mild-to-moderate, transient erythema. No unusual adverse events.	–
Tanaka [38]	Clinical study	20	Japan	Facial skin tightening	Intensif Handpiece (EndyMed Medical, Caesarea, Israel)	3.5 mm	0 to 25 W	25 Non-insulated gold-plated microneedle	30–200 ms	–	1	None	Significant skin tightening on the lower tewo-thirds of the face, patients were either satisfied or very satisfied with the treatment	6 months	Slight transient burning sensation and mild erythema. No major side effects	
Clementoni et al. [39]	Pilot	33	USA	Mild-to-moderate lower facial and neck skin laxity	INFINI^TM^ Microneedle RF System (Lutronic Corp., Goyang, South Korea)	0.5–3.5 mm	Level 1 (2.5 W) to Level 20 (50 W) in 2.5 W increments	Insulated microneedles with exposed active tip (~300 µm)	10–1000 ms	–	3	None	Improvement in facial and neck laxity, GAIS improvement, physician and patient aesthetic improvement scores	6-month, one-year telephone survey	Mild-to-moderate transient pain during treatment, mild edema, Mildly, stippled papules and erythema

The sample sizes ranged from 9 to 126 patients. Almost 37% of the studies focused on atrophic acne scars or acne vulgaris as the primary indication for RFMN treatment. Other indications included periorbital wrinkles, photoaging, skin laxity, enlarged pores, striae distensae, hyperhidrosis, rosacea, facial fine lines, and melasma. Thirty-two studies assessed the exclusive use of RFMN in various conditions, while 7 studies compared RFMN with other modalities such as *Botulinum* toxin and laser.

### Treatment Protocol

Needle depth varied with the treatment site. On the face, depths typically ranged from 0.5 to 3 mm, with lower depths preferred for delicate areas like the periorbital region [11] and higher depths for more atrophic conditions, such as acne scars [19]. Energy settings were similarly heterogeneous, spanning from 5 to 6 W [52] to as high as 50 W [43], often without a clear rationale for the chosen parameters.

For periorbital wrinkles, Lin et al. [11] employed MicroRF9 at 4–10 W and 0.5–1.5 mm depths in two sessions, while Zhang et al. [18] applied INTRAcel at 12.5 W for facial photoaging. By contrast, Ding et al. [19] treated atrophic acne scars with FMR 49 at a deeper penetration of 1.8 mm and an energy of 6–16 W over three sessions. Similarly, Eid et al. [48] reported using Vivace® at 20–25 W and 3.5 mm for axillary hyperhidrosis. These variations suggest that anatomical characteristics and treatment goals influence parameter selection, though few studies explicitly discussed optimization strategies.

Temperature settings were rarely reported; only Suh et al. [31] provided this detail (67 °C for 3 seconds per insertion), highlighting a broader gap that limits reproducibility. The number of sessions ranged from one to six, with three sessions being most common, but again, justification for these protocols was seldom provided. A consolidated parameter framework summarizing optimal depths, energy settings, and needle-type selection across indications is provided separately in Table 4.

**Table 4:** MNRF treatment parameters by indication

Indication/Target	Depth range	Energy/Pulse Profile	Needle selection (Scientific Rationale)	Sessions
Atrophic acne scars	1.5–2.5 mm (up to 3.0–3.5 mm in thick dermis)	5–18 W/50–70 W or 30–98 mJ/pin or 11–142 mJ/pin; 100–600 ms	Insulated needles deliver focused deep-dermal coagulation with epidermal sparing; non-insulated needles are suitable when combined epidermal and superficial dermal remodeling is desired	1–5
Facial laxity / skin tightening	0.5–3.0 mm	5-12 W; 100–600 ms	Insulated needles concentrate RF at the tip for deep collagen remodeling while minimizing epidermal injury and PIH risk	3
Photoaging wrinkles	1.7–2.7 mm	12–17 W; 50–150 ms	Non-insulated needles provide full-length energy delivery, enabling epidermal and papillary dermal resurfacing for fine lines and texture	1–3
Periorbital wrinkles	0.6–1.5 mm (up to 1.8 mm if tolerated)	12.5W or 60 mJ/pin; 100 ms	Non-insulated ultrathin needles allow controlled superficial coagulation in thin periocular skin; insulated needles may be used at low energy to limit epidermal heating	3–4
Neck rejuvenation	1.8–2.5 mm	12–14 W; 80–100 ms	Non-insulated gold-plated needles facilitate uniform epidermal-to-papillary dermal heating suited for thin neck skin	1–3
Rosacea	2.0–2.5 mm	5–12 W; 50–110 ms	Insulated needles are preferred due to selective deep-dermal thermal delivery to vascular targets while sparing epidermis; non-insulated use is reported at lower energies	2–3
Melasma	0.8–1.5 mm	Platform-specific (e.g., 2 MHz SYLFIRM)	Non-insulated stainless-steel needles promote superficial dermal remodeling without excessive depth	3
Striae distensae	0.5–3.5 mm	4–50 W; 300–450 ms	Insulated needles are routinely used to target deep dermal collagen disruption characteristic of striae	4-5
Axillary hyperhidrosis	3.0–3.5 mm	Long pulses: 600 ms (device-dependent power)	Insulated needles, or long-pulse non-insulated needles as demonstrated in randomized controlled trials, effectively coagulate eccrine sweat glands located in the deep dermis	2–3
Acne vulgaris	0.8–2.0 mm/ 1400–1600 mm	~12.5 W; 50–110 ms/400–500 ms	Partially insulated or non-insulated needles provide selective papillary dermal heating appropriate for inflammatory acne pathways	3

### RFMN Efficacy

A summary of efficacy parameters reported in the included studies is presented in Table 5.

**Table 5:** Efficacy and patient-reported outcomes in the included studies

Authors	Device and number of MNRF sessions	Patient-reported outcomes (PROs)	Efficacy summary
Lin et al. [11]	MicroRF9 (Shenzhen Peninsula Medical Co. Ltd., China); 2	All patients experienced pain during MNRF^a^ In terms of perceived improvement, 11.1% felt very much improved, and 55.6% improved.	FWES^b^ scores improved at 6 months (p<0.001); wrinkle depth and indentation significantly reduced at all sites (*p* < 0.05).
Anwer et al. [16]	Intensif (EndyMed Medical, Israel); 1,2, or 3	Mild pain was reported by the patients (Pain score 3–4).	WAS^c^ scores decreased significantly after MNRF^a^; improvement rates after 1 session were 39.13% (25–30 yrs) and 16.39% (> 60 yrs).
Ding et al. [19]	FMR^d^ 49, (Shenzhen Peninsula Medical Co., Ltd, China); 3	Mild to moderate pain; average pain score of 3.4 reported by the patients.	All patients showed ECCA^e^ score reduction post-FMR^d^ (26%-100%). Manchester Scale scores decreased for distortion (2.4 ± 0.6 to 1.6 ± 0.6), color (2.3 ± 0.7 to 1.6 ± 0.6), and VAS^f^ (3.6 ± 1.3 to 2.2 ± 1.1) (all *p* < 0.001).
Navyadevi et al. [21]	Lumenis Legend PRO (Lumenis, Israel); 4	Not reported	GB^g^ qualitative assessment showed patients with Grades 2, 3, and 4 at baseline were 6 (15%), 17 (42.5%), and 17 (42.5%), respectively. At the end of follow-up, patients with Grades 1, 2, 4, and 4 acne scars were 5 (12.5%), 24 (60%), 10 (25%), and 1 (2.5%), respectively, which were statistically significant (*p* < 0.05)
Liu et al. [35]	Gold Microneedle United II (Peninsula, China); 3	The differences in the VAS^f^ scores of the treated sides and control sides were 4.51 ± 1.38, 5.87 ± 1.28, 5.80 ± 1.09 at 1, 3, and 6 months, respectively.	3D volume reduction in lower eyelid, cheek, and mandible at all time points was significant vs. control (*p* < 0.05).
Eid et al. [48]	Vivace® device (Shenb Co., Korea); 3 in RF^h^, 1 in Botox group	Patients reported significantly higher satisfaction with BT-A^i^ than with MNRF^a^ at all follow-ups: *p* ≤ 0.001. Pain was higher with FMR^d^ (mean 6.48) than BT-A^h^(mean 4.00), p<0.001.	At 6 months, the Botox group showed an 84.1% reduction in sweating (MSIT^j^), while the FMR^d^ group showed a 68.2% reduction (*p* = 0.006).
Cheles et al. [13]	TriFractional RF^h^ (Pollogen, Israel); 3	Patients reported mild discomfort (mean VAS^f^ 4.1).71% rated periorbital wrinkles as “improved” or “very much improved,” and 83% reported satisfaction with comfort, recovery, and results.	Aggregated facial LFW^k^ score decreased from 13.00 ± 4.75 to 6.09 ± 3.90 at 3 months post-treatment (reduced to 53%, *p* < 0.05); wrinkle severity significantly reduced in forehead, glabella, periorbital, nasolabial, and mouth corners (*p* < 0.05).
Suh et al. [31]	Profound system (Candela Medical, South Korea); 1	At the 4-month follow-up, 86.7% of patients reported satisfactory results.	At 4 months, objective photographic assessment showed 53.3% of patients were rated as “very much improved,” histological analysis confirmed increased epidermal thickness, new collagen formation, and replacement of elastotic material with organized elastic fibers.
Huang et al. [20]	Bodytite, (Chongqing Peninsula Medical Technology Co. Ltd, China); 3	Patients reported mild, tolerable pain during MNRF^a^ treatment (VAS^f^ 1–4, mean 3.08).	Overall, ECCA^e^ showed from around 40 at baseline, to about 12 at the last visit.
Hendel et al. [22]	Genius RF^h^ (Lutronic, South Korea); 1	Pain and discomfort during treatment were generally reported as moderate to high with MNRF at median 7 (4.5–8.0) vs. CO₂^l^‚ laser (median 5.5 (3.0–6.3), *p* = 0.009. Patients generally reported moderate-to-high satisfaction for both MNRF and laser (3 months),	At 3 months, MNRF^a^ improved skin texture by a median of 1 point (IQR^m^ 1–2) on the assessment scale (*p* < 0.001).
Kim et al. [12]	Genius RF^h^ (Lutronic, South Korea); 3	The pain numeric rating scale (NRS) score, was an average of 5.33. Patient satisfaction varied from very satisfied (4), five satisfied (3), to three were slightly (3).	Fitzpatrick WAS^c^ scores decreased at 8 weeks, 12 weeks, and 6 months (*p* = 0.01).
Gulfan et al. [40]	SYLFIRM (Viol, Korea); 3	Participants tolerated treatment well, with a reported mean pain score of 2.0 ± 1.8 (minimum, 0; maximum, 6).	Significant reduction in MI^n^ (*p* = 0.010 at 1 month, *p* < 0.001 at 2-6 months); EI^o^ decreased at 2 months (*p* = 0.009); skin roughness and mMASI^p^ decreased from 2 weeks to 6 months (*p* = 0.002; *p* < 0.001, respectively).
Wang et al. [41]	Intensif (EndyMed Medical, Israel); 3	The VAS score for pain was very low (average: 2.29 ± 0.82),); the average ^z^DLQI gradually decreased with the number of treatments (mean at visits 0 and 3: 16.70 ± 3.55 and 10.48 ± 2.92, respectively (*p* < 0.001).	CEA^q^ reduction: 2.65±0.59 to 1.56±0.50 (*p* < 0.001); CEA^q^ success: 44.12% (*p* < 0.001); IGA^r^ success: 91.67% (*p* < 0.001).
Nguyen et al. [53]	Genius RF^h^ (Lutronic, South Korea); 1 to 3	Most patients were very (65%) or mainly (95%) satisfied. All patients would recommend the treatment and reported minimal downtime.	Mean submental volume reduction was −4.72 ± 10.07 cm^3^, with significantly greater reduction in patients with moderate-to-severe laxity *p* < 0.0001).
Emam et al. [23]	Vivace Microneedling Device (Vivace, Korea); 4	Downtime was shorter with MNRF^a^ (≤2 days) versus Erbium: YAG (3–7 days). Nine participants were very satisfied with the results on the MRF side, 8 participants were satisfied, and 4 participants were neutral.	Acne scar scores decreased significantly after treatment: MNRF^a^ from 18.14 to 12.05 (−6.10, *p* < 0.001), Erbium YAG from 19.33 to 13.19 (−6.14, *p* < 0.001).
Xiao et al. [33]	Intensif (EndyMed Medical, Israel); 1	Almost all subjects reported 3 to 4 points of pain scores.	Neck aging scores: about 50% subjects achieved ≥ 50% improvement, and average improvements were 37.0% at 3 months follow-up and 41.6% at 6 months follow-up (M6). Seventy.
Dou et al. [17]	INFINITM (Lutronic Co., Korea); 3	At 1- and 3-month post-treatment, 73–80% of MNRF^a^ patients and 66–73% of non-ablative fractional laser patients reported >50% satisfaction with skin laxity.	Skin laxity scores decreased from 2.67 to 0.93 (MNRF^a^) and 2.63 to 0.83 (non-ablative fractional laser).
Nada et al [43]	RF™; (Hanz, Beijing, China); 3	MNRF^a^ treatment (average 4.23) was non significantly considered more painful than Erbium: YAG (average 3.41).	Based on SASIS^s^ scores, with MNRF, 7 patients (41.2%) achieved very good improvement (50%–75% improvement), 6 patients (35.3%) had good improvement.
Rummaneethorn et al. [47]	FMR^d^ DeAge EX® applicator device (Daeshin Enterprise Co., Ltd., South Korea); FMR^d^:2, BT-A^i^: 1	Slightly higher VAS^f^ pain scores for microneedle RF^h^ vs. BT-A^i^ (3.45 vs. 2.65).	Reduction in HDSS^t^ scores was significant in both groups (*p* < 0.001).
Zeng et al. [49]	INTRAcel (Jeisys, Korea); 3	Patient satisfaction was higher for MNRF^a^ after the first treatment (8.1 ± 1.2 vs. 6.4 ± 1.0; *p* < 0.05).	Both MNRF^a^ and FR treatments significantly improved acne severity.
Nilforoushzadeh et al. [34]	INFINI RF^h^ (Lutronic Corp, South Korea); 6	At three months after the last session, patient satisfaction was significant (*p* < 0.05).	TEWL^u^ decreased by 18.44%; skin pores and spots reduced; dermal/epidermal density and thickness increased significantly.
Sobhi et al. [44]	Vivace Microneedling Device (Vivace, Korea); 5	Patient were more satisfied in area B (fractional CO₂^l^ laser), without statistical significance (*p* = 0.409).	MNRF^a^ showed a 44.29% mean reduction in scar width with higher blinded physician scores, while CO₂^l^ laser showed a 36.76% reduction.
Zhang et al. [18]	INTRAcel (Jeisys, Korea); 3	Patients reported lower pain with unipolar RF^h^ (VAS^f^ 3.19 ± 1.77) compared to bipolar RF^h^ (VAS^f^ 5.63 ± 1.78).	GPS^u^ score improved from 2.54 to 1.98 in both 1 and 6 months. GAIS^w^ showed improvement in 93% patients for rhytides, 100% patients for tightening, and 59% for complexion improvement at 1 month.
Elawar et al. [24]	Intensif (EndyMed Medical, Israel); 2-6	The average pain assessment score was 1.5 (0–10). Patients reported overall satisfaction and improvement with the treatment.	Objective assessments using GAIS^w^ showed that 37.5% of patients had 51–75% improvement in acne scars, and 59.37% had 26–50% improvement.
Kwon et al. [50]	Secret (Ilooda, Korea); 3	The average duration of downtime affecting their quality of life was 1.6 ± 1.5 days for FMR side, and 1.5 ± 1.1 days for the diode laser side, which was not significantly different (*p* > 0.05).	Inflammatory acne lesions decreased 58.2% with MNRF^a^ and 39.3% with diode laser(*p* < 0.05).
Faghihi et al. [25]	INFINI RF^h^ (Lutronic Corp, South Korea); 3	Mean patient satisfaction score was 5.1 ± 1.6 for MNRF^a^ without subscision vs.6 ± 2.2 for MNRF with subscision.	The mean clinical improvement scores rated by two dermatologists were 45.6 ± 17.6% for the MNRF^a^ + subcision group and 39.6 ± 14.8% for the MNRF^a^ alone group (*p* = 0.009).
Pudukadan et al. [26]	Intensif (EndyMed Medical, Israel); 3	Patient satisfaction was very high, attributed to minimal treatment discomfort and low downtime.	Improvement of at least 1 acne scar grade was noted in 11 of 19 patients (57.9%) after 1 month and in 9 of 9 patients (100%) who were examined after 3 months.
Lee et al. [14]	INTRAcel (Jeisys, Korea); 3	Mean participant satisfaction score for the procedure was 3.05.	The mean treatment efficacy was—1.075 ± 0.52 (*p* < 0.001) as per WAS^c^ Scores.
Lan et al. [27]	Intensif (EndyMed Medical, Israel); 3	More than 70% patients were satisfied in the fractional MNRF^a^ group.	The mean decrease in ECCA^e^ scores from baseline was 41.33 ± 20.19 on the fractional micro‐plasma RF^h^ side and 32.17 ± 17.35 on the fractional MNRF^a^ side (*p* < 0.05).
Goyal et al. [28]	MicroFrxl (Derma, India); 3	Mean patient satisfaction steadily increased over visits, reaching 1.60 for MNRF^a^ and 1.64 for EL1550 nm laser at the final visit.	Both MNRF^a^ and EL1550 nm laser treatments significantly improved GB^g^ scar grades over the study period (*p* < 0.05).
Park et al. [42]	INFINI RF^h^ (Lutronic Corp, South Korea); 2	Satisfaction scores increased over time, indicating progressive patient-reported improvement in 12 weeks.	Clinical improvement of rosacea was observed in 17 out of 21 patients.
Ovhal et al. [29]	Dermatrix (GSD, India); 3	Nearly all patients reported burning pain lasting 1–2 hours after treatment.	Out of 31 patients, 64% showed a 2-grade improvement in acne scar severity based on the GB^g^ scale. Specifically, 63.8% of patients with grade 3 scars improved to grade 1, and 75% of those with grade 4 scars improved to grade 2.
Swaroop et al. [45]	DERMA INDIA (MR 16-2SB, India); 4	6 out of the 20 patients were ‘very satisfied’ with the treatment, 8 were ‘satisfied’ and 6 were ‘slightly satisfied’.	Out of the 20 patients - 6 patients showed marked improvement, while 12 patients showed moderate improvement.
Lodi et al. [51]	Vivace (Deka M.E.L.A, Calenzano, Italy); 3	Patients experienced no pain or discomfort following the MNRF^a^ treatment and less downtime vs traditional monopolar/bipolar RF^h^ therapy.	Mean GEA^y^ scale scores significantly decreased from 3.80 (− 0.51) at baseline to 0.43 (−0.51) at 6-month (*p* < 0.001) follow-up after the last treatment session.
Ghosh et al. [46]	DERMA INDIA (MR 16-2SB, India); 3	The mean Patient Satisfaction VAS^f^ Score after treatment was 2.1 in Group B (MNRF^a^) as compared to 1.8 in Group A (Fractional CO₂^l^).	The mean Global Improvement Score at the end of 1 month after the last treatment session in Group B (MNRF^a^) was 2.13 compared to 1.86 in Group A (Fractional CO₂^l^), (*p* < 0.05).
Baskan et al. [30]	Secret (Ilooda, Korea); 1–5	Temporary pain was reported by 100% of patients, with the most common VAS^f^ score noted to be 1.	Improvement >25% in 77.7% (physicians' assessment) and 88.9% (patients' assessment).
Gao et al. [15]	High Frequency Electrocautery (United II, Shenzen Peninsula Medical Co.); 2	Pain during treatment was tolerable, except for one patient (out of 25) who dropped out due to pain; the VAS for pain was 4.05 ± 1.26.	1 month: wrinkle count decreased (*p* < 0.5); skin gloss and dermal thickness increased (*p* < 0.5); no significant differences in hydration, TEWL, melanin and pH; 3 months: wrinkle count and TEWL decreased (*p* < 0.5); 6 months: wrinkle count and TEWL continued to decrease, hydration, pH and dermal thickness continued to increase (*p* < 0.5 for all)
Lu et al. [37]	Bodytite (Invasix, Israel); 3	Deep vs. superficial skin penetration was tested. Pain grade was ^f^VAS 3.4 on the deep side and 3.2 on the superficial side (*p* > 0.01).	Overall, MNRF treatment resulted in an improvement of periorbital wrinkles, nasolabial folds, and skin laxity at 12 months. Nasolabial grooves: Mean score on the deep side (3.3 ± 0.9) was significantly higher than on the superficial side (2.0 ± 0.7) for (*p* < 0.05); infraorbital rhytides: mean score at the deep side (3.2 ± 0.8) was slightly higher than on the superficial side (2.8 ± 0.6) (*p* > 0.05).
Gold et al. [36]	Intensif applicator (EndyMed, Caessarea, Israel); 3	The average pain level was mild to moderate (level 4 out of 10). Improvement in100% of patients; 65% of patients had significant improvement (^w^GAIS levels 3–5)	The average Fitzpatrick’s wrinkle scale scores decreased: baseline: 5.04 ± 1.22, 1 month: 3.829 ±1.69, 3 months: 3.5 ± 1.66 (*p* < 0.001)
Tanaka Y [38]	Intensif Handpiece (EndyMedMedical, Caesarea, Israel); 1	Ninety percent of patients were “satisfied” or “very satisfied” on a 5-point scale of 0–4 (average score: 3.53 ± 0.84).	Significant tightening effects observed on the lower two-thirds of the face, cheeks, nasal and peri-oral areas, lasting for 6 months post-treatment
Clementoni et al. [39]	INFINI (Lutronic Corp, Goyang, South Korea).	In global assessment 81.8% of the patients achieved moderate or higher results; 87% of patients were very satisfied or better	Significant post-treatment decrease in the cervicomental and gnathion angles was seen, at 28.58° and 16.68°, respectively (*p *<0.0001 for both).

Abbreviations: ^a^*MNRF* Microneedling radiofrequency; ^b^*FWES* Facial Wrinkle Evaluation Scale; ^c^*WAS* Wrinkle Assessment Scale; ^d^*FMR* Fractional microneedling Radiofrequency; ^e^*ECCA* Échelle d'Évaluation Clinique des Cicatrices d'Acné (Clinical Acne Scar Assessment Scale); ^f^*VAS* Visual Analog Scale; ^g^*GB* Goodman and Baron; ^h^*RF* Radiofrequency; ^i^*BT-A* Botulinum toxin A; ^j^*MSIT* Minor Starch–Iodine Test; ^k^*LFW* Lemperle Facial Wrinkle Score; ^l^*CO₂* Carbon dioxide; ^m^*IQR* Interquartile range; ^n^*MI* Melanin Index; ^o^*EI* Erythema Index; ^p^*mMASI* Modified Melasma Area and Severity Index; ^q^*CEA*: Clinician’s Erythema Assessment; ^r^*IGA*: Investigator Global Assessment; ^s^*SASIS*: Stretched Acne Scar Improvement Scale; ^t^*HDSS* Hyperhidrosis Disease Severity Scale; ; ^u^*TEWL* Trans epidermal water loss; ^v^*GPS* Global patient satisfaction; ^w^*GAIS* Global Aesthetic Improvement Scale; ^x^*NAFL* Non-ablative fractional laser; ^y^*GEA* Global evaluation of acne; ^z^*DLQI* Dermatology Life Quality Index

#### Atrophic Acne Scars

Among the 41 studies analyzed, 11 studies (including >500 patients) assessed RFMN for atrophic acne scars. RFMN was consistently effective across all the studies, suggesting a 26–100% decrease in ECCA (échelle d'évaluation clinique des cicatrices d'acné) scores [19], a 34.35% decrease in Goodman and Baron grading (GBG) scores [21], and a ≥ 2-grade improvement in GBG scores in 64% of patients [29]. Split-face trials reported comparable efficacy to fractional lasers, with some indicating faster recovery and fewer pigmentary changes with RFMN [23, 28]. Combination therapies, particularly with subcision, yielded greater improvements than RFMN alone [25].

#### Skin Laxity and Wrinkles

Thirteen studies assessed RFMN for skin laxity and wrinkles, and all reported improvements; however, the findings should be interpreted cautiously. Nilforoushzadeh et al. [34] reported an 18.44% decrease in transepidermal water loss (TEWL), alongside increased skin elasticity (R7), dermal density, and dermal thickness. Gao et al [15] also reported significant reduction in wrinkle count and TEWL and increased hydration and dermal thickness. In a study by Nguyen et al. [53], RFMN treatment resulted in a significant decrease in submental volume, with a greater benefit observed in patients who received more sessions and those with moderate-to-severe laxity. In another study, nasolabial groove volume reduced from 0.47 to 0.33 mm^3^ [17]. These results, corroborated by VISIA and 3D imaging and supported by histologic evidence of increased collagen and elastin, suggest a positive trend.

#### Other Indications

RFMN treatment led to measurable improvement in several other skin conditions, including periorbital wrinkling and photoaging, as well as rosacea, striae distensae, and melasma. However, there was some variability in the magnitude of benefit among these skin conditions.

For skin laxity and photoaging, several studies reported consistent and sometimes dramatic benefits with RFMN treatment. Lu et al. [37] studied the effects of deep vs. superficial penetration of microneedles for facial skin and reported significant improvement in periorbital wrinkles, nasolabial folds, and skin laxity at 12 months after 3 sessions of MNRF treatment. Anwer et al. [16] observed modest improvement with a 25% reduction in wrinkle severity, while Gold et al. (Gold et al) reported a significant decrease in Fitzpatrick’s wrinkle scale scores (baseline: 5.04 ± 1.22, 1 month: 3.829 ± 1.69, 3 months: 3.5 ± 1.66 (*p* < 0.001)). Xiao et al. [33] reported a striking 75% improvement in neck aging scores; Tanaka [38] reported significant tightening effects on the lower face, cheeks, nasal and peri-oral areas, which sustained for 6 months. Neck laxity was similarly reported as improved by Clementoni et al [39], with a significant lowering of cervicomental and gnathion angles (*p* < 0.0001 for both). Similarly, Lin et al. [11] found significant improvements in validated wrinkle scales, and Kim et al. [12] provided histological evidence of new collagen deposition, a biological signal that reinforces the clinical findings. Lee et al. [14] reported meaningful wrinkle reduction with RFMN treatment in darker skin types. All the included studies reported consistent efficacy in skin tightening and rejuvenation across diverse populations and Fitzpatrick skin types, thereby suggesting RFMN has good effectiveness for skin rejuvenation across ethnic groups.

Among studies of RFMN applied for acne vulgaris, Lodi et al. [51] reported a significant reduction in Global Evaluation Acne (GEA) scores and Zeng et al. [49] reported faster lesion reduction and superior efficacy compared to RF. Kwon et al. [50] reported that RFMN outperformed diode laser treatment for long-term acne control. In the study on rosacea by Park et al. [42], 17 of 21 treated patients with Fitzpatrick skin types III–V showed clinical improvement and histologic improvement after two sessions. For striae distensae, the results for RFMN treatment were mixed. While Ghosh et al. [46] found RFMN to be more effective than the CO₂ laser, Sobhi et al. [44] found both RFMN and the CO_2_ laser to be equally effective. Swaroop et al. [45] reported that RFMN was safe and effective for striae distensae, with 18 out of 20 patients showing marked or moderate improvement.

### Safety

**Fig. 4:**
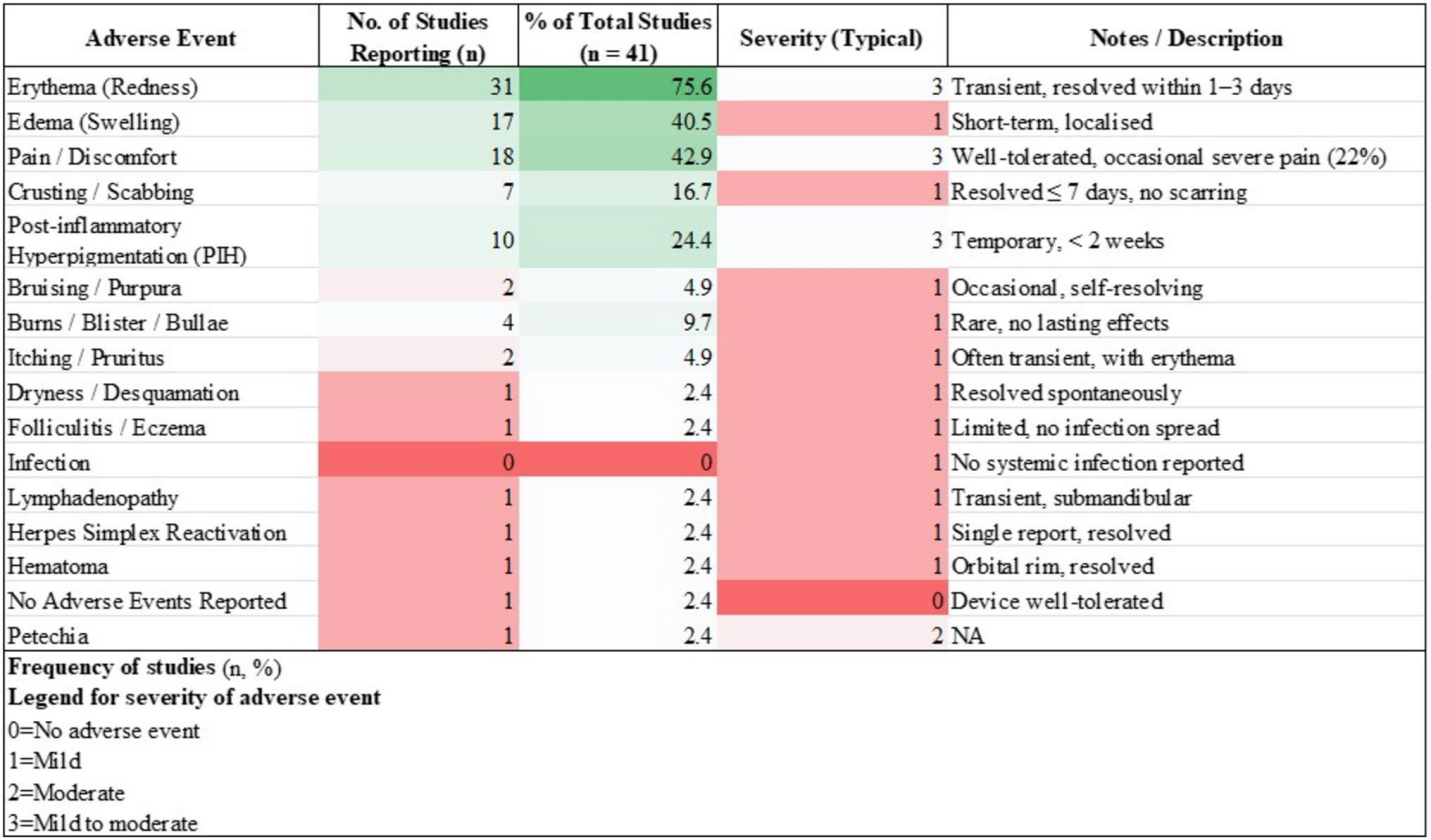
Heat map of frequency and severity of adverse events. The figure presents the adverse events recorded in the included studies. The % of total studies that reported each adverse event is represented as a number, and the intensity of color represents the extent to which that adverse event was seen (increased occurrence means a darker color). The severity of the adverse event is also presented as a severity score 0 representing no adverse event; 1.0 represents mild, and 2.0 represents moderate level of severity

The heat map in Figure 4 presents a summary of all safety parameters and reported AEs. RFMN was demonstrated to be an overall safe procedure, with minor AEs in the majority of cases; AEs were also temporary and relatively easily resolved. In all studies on atrophic acne scars, self-limiting erythema was reported, which faded in 24–72 hours [19, 21, 30]. Edema was reported frequently [19, 26], occurring in 30–65% of patients. Crusting [30] and post-inflammatory hyperpigmentation (PIH) occurred infrequently [26, 28]. Pain was reported across all studies; however, a significant variance was observed (VAS 1.5 to 8.8) [23, 24].

In studies on photoaging and periorbital/neck wrinkles, the AEs, which included erythema, edema, and occasional pinpoint bleeding, were similarly mild, transient, and resolved within 1–7 days. Long-term complications such as scarring, dyspigmentation, or worsening of melasma were consistently absent [14, 18]. Zeng et al. found RFMN had lower pain scores (4.3 vs. 6.7) than fractional RF. Although most studies reported only mild-to-moderate pain, Zhang et al. [18] reported a trend toward greater discomfort with bipolar mode compared to unipolar mode. However, the absence of standardized pain assessment tools and inconsistent reporting across studies limits the strength of these observations.

RFMN was noticeably safer than CO₂ laser treatment, with a favorable side effect profile in cases of striae distensae [46]. PIH was conspicuously absent with RFMN and observed only in patients treated with the fractional CO₂ laser [44]. In patients with rosacea, only mild pain and transient erythema were reported with RFMN treatment [42, 51]. Overall, RFMN was consistently well-tolerated with minimal downtime and low risk of pigmentary changes. In October 2025, the US FDA issued a safety communication noting rare but serious complications associated with certain dermatologic and aesthetic uses of RFMN, emphasizing appropriate use, practitioner training, and ongoing surveillance. The evidence in this review indicates that treatment delivered by appropriately trained and certified providers, with adherence to indication-specific parameters and anatomical tailoring of energy and needle depth, mitigates these risks, with most studies reporting only mild, transient adverse events and no serious complications.

### Patient-Reported Outcomes (PROs)

**Fig. 5:**
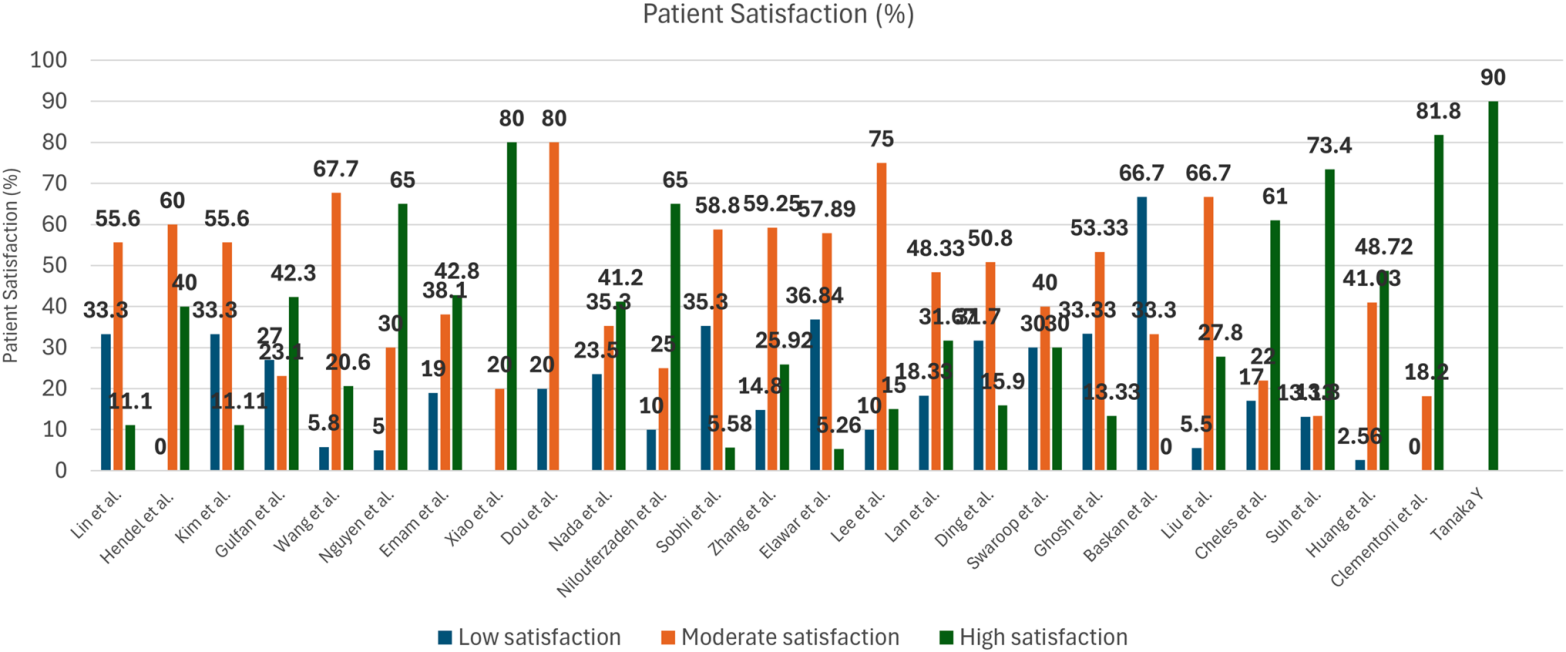
Patient-reported satisfaction in the included studies. The graph shows the proportion of patients (% of total) in study and the satisfaction level reported, categorized as low, moderate or high

PROs were evaluated in most studies (26 of 41 studies; 63.5%), and the results showed that most patients were satisfied with RFMN. Figure 5 illustrates the variations in patient satisfaction, ranging from very satisfied to moderately satisfied. Pain scores ranged from 3 to 5.6/10, and downtime was minimal, although RFMN had a longer crusting duration than laser treatment. Overall, patients reported that they found visible improvements and experienced minimal discomfort with RFMN treatment. However, *Botulinum* toxin (studied in PAH) outperformed RFMN in terms of pain and tolerability [13, 47].

### Device Comparison

Various RFMN devices were investigated across the included studies with most reporting positive efficacy and safety outcomes. However, the evidence base remains heterogeneous, and direct comparisons between devices are limited by differences in patient selection, outcome measures, and follow-up durations.

A summary of the devices used is presented in Supplementary Table 3. The Intensif system (EndyMed, Israel) was the most extensively studied, evaluated for indications ranging from acne scars and neck rejuvenation to wrinkles, skin laxity, and rosacea [16, 24, 28, 33, 36, 38, 54]. Its motorized needle delivery system, cited as enabling smoother insertions and lower pain scores [24], highlights a design-driven advantage; however, standardized pain and satisfaction metrics were seldom employed, limiting the reproducibility of these findings.

The INTRAcel device (Jeisys Med Inc, Korea), equipped with gold-plated insulated needles, was applied in three studies for acne, periorbital, and facial wrinkles [14, 18, 49]. Zhang et al. [18] reported that this design preserved energy while minimizing epidermal trauma, correlating with shorter downtime and sustained improvements up to six months. Although promising, these observations are based on small cohorts and require larger, controlled evaluations.

Similarly, the Infini MFR system (Lutron, South Korea) was used in five studies targeting rosacea, acne scars, skin tightening, and photoaging [17, 25, 34, 39, 42]. Reported benefits were consistent across indications, though hampered by variation in protocols and endpoints.

The FMR49 needle head (Shenzhen Peninsula Medical Co., Ltd., China) demonstrated a unique negative-pressure suction feature to displace eyelid skin during periorbital treatments, offering a safety advantage [11].

Finally, the Lumenis Legend Pro (Lumenis, Israel), which was applied in two studies [21, 46], was reported to improve skin scars, even in darker skin types (Fitzpatrick III–IV), and was described as suitable for use in combination with other modalities. While these device-specific innovations show promise, the lack of head-to-head comparative data limits the ability to determine relative efficacy or safety advantages.

## Discussion

The current evidence is encouraging; RFMN has shown potential benefits across diverse skin types and conditions. However, most studies are small, short in duration, and methodologically heterogeneous, which may mean that the data remain inconclusive. While RFMN shows promise in addressing texture, scarring, pigmentation, and laxity, the current evidence base is not yet strong enough to declare it a universal solution. Variability in study design, outcome measures, and reporting quality necessitates a cautious interpretation of these promising findings.

At a mechanistic level, RFMN works by using radiofrequency energy to disrupt the hydrogen bonds in dermal collagen, causing immediate tissue contraction followed by a wound-healing response that induces further tightening [55, 56]. While higher energy levels have been shown to correlate with better outcomes [3], they must be tailored to the anatomical region to avoid complications. In a study, Dayan et al. [57] recommended using 15–30 V in the periorbital area and 20–40 V in the neck region, stating that these settings prevent adverse effects such as skin redness, hash marks, and pain.

Beyond applying a broad range of energy settings, temperature control and cooling strategies are critical, yet underreported. Dermal temperatures of 65–70 °C are believed to optimize collagen remodeling, but epidermal injury can occur at temperatures above 44 °C [5]. A few studies described post-procedure cooling with saline gauze draping for 15 min, application of cooled air, and a cooling mask [11, 37, 49]. Interestingly, Suh et al. [31] were the only group to include a few details regarding temperature settings. They reported using a single-pass treatment applied to the dermis at a temperature setting of 67 °C for 3 seconds. The absence of standardized temperature and cooling data limits both reproducibility and safety assessments.

Several randomized controlled trials have demonstrated that RFMN is associated with significant improvements in periorbital rhytids, acne scars, and overall skin rejuvenation. A 2025 systematic review pooling 16 studies (481 patients) concluded that RFMN effectively improves acne scarring [58], and a single RCT reported about a 52% reduction in disease severity [59]. Additionally, dose–response studies linked higher RF energy to greater histological and volumetric improvements [32]. Nevertheless, improvements of 20–60% were reported in facial wrinkling, texture, and laxity [56]. The benefit appears to be robust across different anatomical sites and for both monopolar and bipolar radiofrequency modalities [3, 20]. Similarly, the studies included in the current review reported high efficacy rates for RFMN in skin rejuvenation (periorbital wrinkles, skin laxity, facial wrinkling, etc.). Reported effect sizes ranged from a 25% reduction in wrinkle scores [16] and GAIS score (facial wrinkle) improvement rate of 100% [36] to 100% in ECCA scores [19], suggesting that patient selection, protocols, and outcome metrics drive much of the variability.

Evidence comparing RFMN with fractional lasers for acne scars and striae suggests that the two modalities offer broadly comparable efficacy. Four RCTs [23, 28, 43, 44] reported no statistically significant differences between the approaches, reinforcing the idea that RFMN is not necessarily superior but is a viable alternative. However, two smaller studies indicated modest advantages with RFMN. Ghosh et al. [46] observed improved outcomes in striae, while Kwon et al. [50] reported greater reductions in acne lesion counts. These findings, while encouraging, are limited by small sample sizes and variations in study design.

Importantly, Reddy et al. [60] highlighted that RFMN’s primary advantages over lasers lie in its more favorable safety and recovery profile, particularly shorter downtime and fewer pigmentary changes, rather than in superior efficacy. This is clinically relevant, especially for patients with darker skin types or those requiring a faster return to routine activities. Overall, the current evidence positions RFMN as a credible alternative to established fractional laser therapies rather than a direct replacement, necessitating individualized treatment planning based on patient characteristics and preferences.

For primary axillary hyperhidrosis, RFMN fared less well against the established gold standard. Two trials comparing RFMN with intradermal *Botulinum* toxin A found that the toxin provided greater symptom reduction, higher patient satisfaction, and less procedural pain [13, 47]. Given *Botulinum* toxin’s long-standing efficacy and durability [61–63]. RFMN currently appears to be a secondary option for patients who cannot tolerate injections. Most studies reported high patient satisfaction (65–95% very or mainly satisfied) and minimal downtime [32, 34]. A recent scoping review also echoed these findings [64]. However, these surveys often lacked validation and may be influenced by self-selection bias, as cosmetic patients are predisposed to rationalize their investment. Without standardized quality-of-life instruments and blinded assessments, patient-reported benefits should be interpreted with caution.

RFMN was generally well-tolerated across diverse skin types. Pain, erythema, and mild edema were the most common adverse events, while PIH occurred in a small minority (2.9–5%) of patients [19, 21]. Larger reviews confirm that such events are usually mild and resolve quickly and spontaneously [5, 56]. However, the short follow-up periods, often only weeks or months, and overrepresentation of Fitzpatrick types II–IV limit the generalizability of these safety conclusions. Longer-term studies involving darker skin types are needed to fully assess the risks of dyschromia, scarring, and other delayed complications. In addition, the trend toward greater discomfort with bipolar modes suggests that the energy delivery pattern in bipolar systems may result in more profound or more concentrated thermal effects.

### Limitations

This review has many limitations. The included studies were heterogeneous with respect to skin indications, and thus, the comparative analysis may not have been optimal. Many of the studies did not report on some important technical details of the RFMN treatment (such as temperature, pulse width, cooling), and most did not describe in detail or analyze the RFMN device being used. This precluded a proper assessment of the impact of the technical parameters on outcomes, as well as a comprehensive comparison of the different RFMN devices. Additionally, methodological rigor was lacking in some studies such as the use of standardized PRO instruments and blinded assessments; additionally, statistical analysis was not consistently performed. This reduces the robustness of the evidence. Reporting bias, including selective outcome reporting, may influence the evidence base for RFMN efficacy. In this study, such reporting bias was mitigated by conducting comprehensive searches across multiple databases and applying transparent inclusion criteria. However, the potential for residual reporting bias is acknowledged as a limitation.

To aid future studies in this area, we have created a RFMN reporting checklist including the items that would standardize comprehensive reporting across studies so that comparisons of devices, treatment regimens, and outcomes can be made across studies. This checklist has been included as Supplementary Table 4.

## Conclusion

This review summarizes recent evidence regarding safety, efficacy, and PROs pertaining to RFMN treatment for various skin indications. RFMN demonstrates credible clinical effectiveness, a favorable safety profile, and high patient satisfaction across a broad range of dermatological indications, particularly for atrophic acne scars and skin laxity. However, evidence strength varies substantially by condition, and long-term comparative data remain limited. The strongest and most consistent evidence observed in the treatment of for photoaging, acne scars, striae distensae, and axillary hyperhidrosis. Moderate evidence was observed for periocular/periorbital wrinkles, acne vulgaris, and rosacea, reflecting limited replication and heterogeneous study designs. Evidence was considered weak for melasma due to sparse data and reliance on single or pilot studies. Certain limitations regarding the strength and quality of evidence across indications, long-term data, study heterogeneity, inconsistent reporting of technical parameters and device characteristics and varying methodological rigor remain. Nevertheless, this study shows that RFMN is a safe, well-tolerated, and effective treatment option for benefit across skin conditions Future studies should incorporate uniform outcome measures, more extended follow-up periods, and rigorous head-to-head comparisons to establish optimized, evidence-based treatment frameworks.

## Supplementary Information

Below is the link to the electronic supplementary material.

Supplementary file1 (DOCX 22 KB)

Supplementary file2: Domain-specific assessment results based on the CASP checklist

Supplementary file3 (DOCX 40 KB)

Supplementary file4 (DOCX 24 KB)

Supplementary file5 (XLSX 20 KB)

Supplementary file6 (XLSX 19 KB)
