# Supplementary material for: Effectiveness of Radiofrequency Microneedling in the Treatment of Dermatological Conditions: A Systematic Review
Source: Aesthetic Plast Surg. 2026 Apr 28;50(13):5282–317. doi: 10.1007/s00266-026-05834-y (PMC13433423; doi:10.1007/s00266-026-05834-y)
Supplement: Supplementary file 1 [file 266_2026_5834_MOESM1_ESM.docx]

Supplementary Table 1. Search strategy and details of queries

| Database | Search String | Limits Applied | Date of Last Search |
| --- | --- | --- | --- |
| PubMed | ("microneedling radiofrequency" OR "radiofrequency microneedling" OR "fractional radiofrequency" OR “fractional RF microneedling” OR “radiofrequency needling”) AND ("derm*)) | English; Humans; 2015–2025 | 23-Jun-25 |
|  | ("microneedling radiofrequency" OR "radiofrequency microneedling" OR "fractional radiofrequency") AND ("dermatol*) |  | 23-Jun-25 |
|  | (("Patient Outcome Assessment") OR (Patient Reported Outcomes) OR ("Efficacy") OR ("Adverse") OR ("Safety")) AND (Radiofrequency Microneedling) OR ("Radiofrequency Resurfacing")) |  | 23-Jun-25 |
| Google Scholar | ("microneedling radiofrequency" OR "radiofrequency microneedling" OR "fractional radiofrequency")) AND ("dermatolog*") | English; Humans; 2015–2025; inclusion of first 600 results | 23-Jun-25 |
|  | (("Patient Outcome Assessment") OR (Patient Reported Outcomes) OR ("Efficacy") OR ("Safety")) AND ((Radiofrequency Microneedling) OR ("Radiofrequency Resurfacing")) AND "derm*" |  | 23-Jun-25 |
| Semantic Scholar | "microneedling radiofrequency" "radiofrequency microneedling" "fractional radiofrequency" "derm* | English; Humans; 2015–2025 | 23-Jun-25 |
|  | “Patient Reported Outcomes” "Efficacy" "Adverse" "Safety" “Radiofrequency Microneedling" "Radiofrequency Resurfacing" |  | 23-Jun-25 |
