# Supplementary material for: Effectiveness of Radiofrequency Microneedling in the Treatment of Dermatological Conditions: A Systematic Review
Source: Aesthetic Plast Surg. 2026 Apr 28;50(13):5282–317. doi: 10.1007/s00266-026-05834-y (PMC13433423; doi:10.1007/s00266-026-05834-y)
Supplement: Supplementary file 2 [file 266_2026_5834_MOESM3_ESM.docx]

Supplementary Table 3. Devices used in the included studies

| Title | Authors | Year of publication | Device/Brand |
| --- | --- | --- | --- |
| Therapeutic Effectiveness of Microneedling Radio Frequency in Different Areas of Periorbital Static Wrinkles: A Self-Controlled Study | Lin F, Song J, Hua Y, Pan L, Guo Y, Hu G, and Yang B. | 2024 | MicroRF9, (Model: United, Shenzhen Peninsula Medical Co. Ltd.) |
| A retrospective study of non-insulated microneedle radiofrequency on wrinkles of facial photoaging subjects | Anwer W, Sun Y, Wang B, Wu Y, and Xiao B. | 2024 | Intensif Handpiece, EndyMed Medical, Caesarea, Israel |
| Efficacy and safety of fractional microneedle radiofrequency for atrophic acne scars: A real-world clinical study of 126 patients | Ding Z, Guo Y, Guo Y, Tang Y, Yin X, Hua H, Zhou Z, and Zhou B. | 2024 | FMR 49, Shenzhen Peninsula Medical Co., Ltd |
| Efficacy and safety of microneedling radiofrequency in acne scars | Navyadevi U, Ganni S, Satya S, Konala S, Kolalapudi SA, Chilka SP, and Anargha B. | 2024 | Lumenis legend PRO Machine, with non-insulated needle tips GEN6, GEN6L; Israel |
| Facial tightening using a novel vacuum-assisted microneedle fractional radiofrequency system: A prospective, randomized, split-face study | Liu Y, Yu W, Zhu J, Cen Q, Zhou L, Ma G, Shang Y, Han Y, and Lin X. | 2024 | Gold Microneedle  United II; Peninsula, Shenzhen, China |
| Long-term efficacy of fractional microneedle radiofrequency versus botulinum toxin-A in primary axillary hyperhidrosis: a randomized controlled trial | Eid RO, Shaarawi E, Hegazy RA, and Hafez V. | 2024 | Vivace® device (Shenb co., Seoul, Korea) |
| Microneedling With RF-Assisted Skin Penetration Improves the Hard-to-Treat Periorbital Wrinkles: Nonrandomized Clinical Trial | Cheles D, Vinshtok Y, and Gershonowitz A. | 2024 | Voluderm MNRF (Pollogen, Israel) |
| Clinical and histological evaluation of microneedle fractional radiofrequency treatment on facial fine lines and skin laxity in Koreans | Suh DH, Cho M, Kim HS, Lee SJ, Song KY, and Kim HS. | 2022 | Profound system (Candela Medical) |
| Efficiency and safety of microneedling fractional radiofrequency in the treatment of Chinese atrophic acne scars: A retrospective study of 3 consecutive treatments with 1-month intervals | Huang L, Liu Y, Fang W, Liu L Sun Q, Lin X, Xu H, and Yang Y. | 2022 | Microneedling Fractional Radiofrequency Bodytite, Chongqing Peninsula Medical Technology Co. Ltd. |
| Fractional CO(2) -laser versus microneedle radiofrequency for acne scars: A randomized, single treatment, split-face trial | Hendel K, Karmisholt K, Hedelund L, and Haedersdal M. | 2023 | Genius; Lutronic Corp |
| Periorbital Skin Rejuvenation of Asian Skin Using Microneedle Fractional Radiofrequency | Kim KE, Park JH, Seul TW, Kim IH, and Ryu HJ. | 2023 | GENIUS®; Lutronic |
| Efficacy and Safety of Using Noninsulated Microneedle Radiofrequency Alone Versus in Combination with Polynucleotides for the Treatment of Melasma: A Pilot Study | Gulfan MCB, Wanitphakdeedecha R, Wongdama S, Jantanapornchai N, Yan C, and Rakchart S. | 2022 | SYLFIRM; Viol, Kyunggi, Korea |
| Efficacy and safety of non-insulated fractional microneedle radiofrequency for treating difficult-to-treat rosacea: a 48-week, prospective, observational study | Wang B, Deng YX, Li PY, Yan S, Xie HF, Li J, and Jian D. | 2022 | Intensif applicator, EndyMed, Caesarea, Israel |
| Radiofrequency Microneedling for Skin Tightening of the Lower Face, Jawline, and Neck Region | Nguyen L, Blessmann M, Schneider SW, and Herberger K. | 2022 | Genius; LutronicMedical Systems, Hamburg, Germany |
| Split-face comparative study of fractional Er:YAG laser versus microneedling radiofrequency in treatment of atrophic acne scars, using optical coherence tomography for assessment | Emam AAM, Nada HA, Atwa MA, and Tawfik NZ. | 2021 | Vivace, Seoul, Korea |
| A retrospective study of neck rejuvenation using a noninsulated microneedle radiofrequency in Chinese subjects | Xiao B, Jiang Y, Wang B, Hu Y, Sun Y, Wu Y, and Qiao S. | 2020 | Intensif Handpiece, EndyMed Medical, Caesarea, Israel |
| Fractional microneedle radiofrequency device and fractional erbium-doped glass 1,565-nm device treatment of human facial photoaging: a prospective, split-face, random clinical trial | Dou W, Yang Q, Yin Y, Fan X, Yang Z, Jian Z, Zhu Y, Wei J, Jing H, and Ma X. | 2021 | INFINITM, Lutronic Co., Goyang-si,  Korea |
| Optical Coherence Tomography-Assisted Evaluation of Fractional Er:YAG Laser Versus Fractional Microneedling Radiofrequency in Treating Striae Alba | Nada HA, Sallam MA, Mohamed MN, and Elsaie ML. | 2021 | RF™; Hanz, Beijing, China |
| A comparative study between intradermal botulinum toxin A and fractional microneedle radiofrequency (FMR) for the treatment of primary axillary hyperhidrosis | Rummaneethorn P and Chalermchai T. | 2020 | FMR DeAge EX® applicator device (Daeshin  Enterprise Co., Ltd., Guro-Gu, and Seoul, South Korea) |
| A split-face comparison of a fractional microneedle radiofrequency device and fractional radiofrequency therapy for moderate-to-severe acne vulgaris | Zeng R, Liu Y, Zhao W, Yang Y, Wu Q, Li M, and Lin T. | 2020 | INTRAcel; Jeisys |
| Biometric changes of skin parameters in the use of microneedling fractional radiofrequency for skin tightening and rejuvenation facial | Nilforoushzadeh MA, Alavi S, Heidari-Kharaji M, Hanifnia AR, Mahmoudbeyk M, Karimi Z, and Kahe F. | 2020 | INFINI, LUTRONIC Company |
| Comparative study between the efficacy of fractional micro-needle radiofrequency and fractional CO(2) laser in the treatment of striae distensae | Sobhi RM, Mohamed IS, Sharkawy DA, and Wahab MAEFA. | 2019 | VIVACE |
| A Prospective Study of the Safety and Efficacy of a Microneedle Fractional Radiofrequency System for Global Facial Photoaging in Chinese Patients | Zhang M, Fang J, Wu Q, and Lin T. | 2018 | INTRAcel (Jeisys, Korea) |
| Non-insulated Fractional Microneedle Radiofrequency Treatment with Smooth Motor Insertion for Reduction of Depressed Acne Scars, Pore Size, and Skin Texture Improvement: A Preliminary Study | Elawar A, and Dahan S. | 2018 | EndyMed Intensif |
| Novel device-based acne treatments: comparison of a 1450-nm diode laser and microneedling radiofrequency on mild-to-moderate acne vulgaris and seborrhoea in Korean patients through a 20-week prospective, randomized, split-face study | Kwon HH, Park HY, Choi SC, Bae Y, Jung JY, and Park GH. | 2018 | Secret, Ilooda, Suwon, Korea |
| Efficacy of fractionated microneedle radiofrequency with and without adding subcision for the treatment of atrophic facial acne scars: A randomized split-face clinical study | Faghihi G, Poostiyan N, Asilian A, Abtahi-Naeini B, Shahbazi M, Iraji F, Fatemi Naeini F, and Nilforoushzadeh MA. | 2017 | INFINI (Lutronic, Korea) |
| Treatment of Acne Scars on Darker Skin Types Using a Noninsulated Smooth Motion, Electronically Controlled Radiofrequency Microneedles Treatment System | Pudukadan D. | 2017 | EndyMed PRO platform with Intensif microneedling handpiece (EndyMed Medical Ltd, Israel) |
| Treatment of periorbital wrinkles with a novel fractional radiofrequency microneedle system in dark-skinned patients | Lee SJ, Kim JI, Yang YJ, Nam JH, and Kim WS. | 2015 | INTRAcel, Jeisys, Korea |
| Comparison of Fractional Micro-Plasma Radiofrequency and Fractional Microneedle Radiofrequency for the Treatment of Atrophic Acne Scars: A Pilot Randomized Split-Face Clinical Study in China | Lan T, Tang L, Xia A, Hamblin MR, Jian D, and Yin R. | 2021 | Intensif Handpiece, EndyMed Medical, Caesarea, Israel |
| Comparison of 1550 nm Erbium Laser and Micro-Needling Radiofrequency for the Treatment of Atrophic Facial Acne Scars: A Prospective, Open-Label, Hospital-based Study | Goyal S, Gahalaut P, and Singh A. | 2024 | MicroFrxl, Derma India |
| Clinical and histological effect of fractional microneedling radiofrequency treatment on rosacea | Park SY, Min S, Kwon HH, Suh DH, and Yoon JY. | 2016 | INFINI; Lutronic, Goyang, South Korea |
| Efficacy of fractional radiofrequency in management of acne scars: A Retrospective study | Ovhal A and Ovhal D. | 2022 | Dermatrix by GSD |
| Evaluation of efficacy and safety of microneedling fractional radiofrequency (MNRF) in the treatment of striae distensae | Swaroop MR, Avik G, Yogesh D, Shale KSM, Mithila R, Krishn Zigu S, Rashmi RM, and Yashwanth R. | 2020 | DERMA INDIA MR 16-2SB |
| Microneedling Radiofrequency for Acne Vulgaris in Adolescents as a Promising Device-Based Treatment: A Clinical Case Series | Lodi G, Fusco I, Zingoni, Tiziano, Cannarozzo G, Sannino, and Mario. | 2025 | Vivace; Deka M.E.L.A, Calenzano, Italy |
| Comparative study of efficacy and safety of fractional CO2 laser and microneedling fractional radiofrequency (MNRF) in the treatment of striae distensae | Ghosh, Avik R, Swaroop M, Ravindranath, M, and Mallya R. | 2020 | DERMA INDIA  MR 16-2SB |
| Evaluation of the efficacy of microneedle fractional radiofrequency in Turkish patients with atrophic facial acne scars | Baskan E, Bulbul, Belli A, and Akin. | 2019 | Secret 2.0; Ilooda, Suwon-si, Korea |
| Treatment of periorbital aging with negative pressure fractional microneedle radiofrequency: A self-controlled clinical trial | Gao Y, Che Q, He Q, Wang W, Luo W, Tong Y, Liu L, Tang Q, Xie Y, Zhou Z, and Wa Q | 2023 | United II, Shenzhen Peninsula Medical Co. |
| Curative effects of microneedle fractional radiofrequency system on skin laxity in Asian patients: A prospective, double-blind, randomized, controlled face-split study | Lu W, Wu P, Zhang Z, Chen J, Chen X, and Ewelina B | 2017 | Bodytite, Invasix, Israel |
| Non-insulated smooth motion, micro-needles RF fractional treatment for wrinkle reduction and lifting of the lower face: International study | Gold M, Taylor M, Tothaus K, and Tanaka Y | 2016 | Intensif applicator; EndyMed, Caesarea, Israel |
| Long-term three-dimensional volumetric assessment of skin tightening using a sharply tapered non-insulated microneedle radiofrequency applicator with novel fractionated pulse mode in Asians | Tanaka Y | 2015 | Intensif Handpiece, EndyMed Medical, Caesarea, Israel |
| Fractional high intensity focused radiofrequency in the treatment of mild to moderate laxity of the lower face and neck: A pilot study | Clementoni MT and Munavalli GS | 2016 | INFINI^TM^ , Kutronic Corp., Goyang, South Korea |
