# Supplementary material for: Effectiveness of Radiofrequency Microneedling in the Treatment of Dermatological Conditions: A Systematic Review
Source: Aesthetic Plast Surg. 2026 Apr 28;50(13):5282–317. doi: 10.1007/s00266-026-05834-y (PMC13433423; doi:10.1007/s00266-026-05834-y)
Supplement: Supplementary file 3 [file 266_2026_5834_MOESM4_ESM.docx]

Supplementary Table 4. MNRF reporting checklist

| Item type | Checklist item |
| --- | --- |
| Patient Data | Skin type (Fitzpatrick Scale I-VI) |
|  | Relevant medical history |
|  | Prior aesthetic treatments |
|  | Primary facial concerns |
| Device data | Device/platform used |
|  | Exact model/version of the device used |
|  | Face tip type (insulated/non-insulated; number of needles) |
| Procedure-specific data | Any standardized skin prep |
|  | Details of any anaesthesia used |
|  | Energy settings used |
|  | Needle depth |
|  | Temperature |
|  | Cooling during procedure |
|  | Treatment duration |
|  | Was the full face treated? Yes/No |
|  | Number of passes per area |
|  | Treatment areas |
|  | Cooling post-procedure or post-care instructions |
| Overall treatment details | Number of sessions |
|  | Interval between sessions |
|  | Follow-up details |
| Physician feedback | Device-specific comments by physician |
| Efficacy - physician & patient-reported | Physician-collected efficacy data (specific to the skin condition, such as wrinkle reduction, pigmentation index, etc.) |
|  | Patient-reported skin condition-specific outcomes (such as pain, satisfaction, downtime, improvement level, etc) collected using standardized instruments |
| Adverse Events | Adverse events (if any) |
|  | Management of adverse events (if any) |
| Photographic documentation | Two or three representative pre-treatment photos (baseline) |
|  | Representative post-treatment photos (day 7, day 30, day 90-if available) |
|  | Any additional follow-up photos (representative) |
